# Tumor cell–derived extracellular vesicles foster the immunosuppressive landscape of pancreatic cancer

**DOI:** 10.1172/JCI197733

**Published:** 2026-07-02

**Authors:** Zainab Hussain, Claudio Montenegro, Christopher Rovera, Djamila Belghoula, Sarah Simha Tubiana, Pascal Finetti, Eugenie Lohmann, Magda Rodrigues, Thomas Bertran, Ghislain Bidaut, Daniel Isnardon, Sophie Vasseur, Francois Bertucci, Stephane Audebert, Luc Camoin, Moacyr Rego, Richard Tomasini

**Affiliations:** 1 Cancer Research Center of Marseille (CRCM), Aix-Marseille University, INSERM U1068, CNRS UMR7258, Institute Paoli-Calmettes, Marseille, France.; 2 Therapeutic Innovation Center, Federal University of Pernambuco, Recife, Brazil.; 3 Department of Medical Oncology, Institute Paoli-Calmettes, Marseille, France.

**Keywords:** Cell biology, Gastroenterology, Immunology, Cancer, Extracellular matrix, Monocytes

## Abstract

Pancreatic cancer remains a devastating disease with limited therapeutic options. Accumulating evidence shows that cancer-associated fibroblasts (CAFs) and tumor-associated macrophages, the predominant cells in the pancreatic cancer (PDAC) tumor microenvironment, hinder antitumor immunity. However, the role of extracellular vesicles (EVs) in such a process is poorly understood. In this study, using human bone marrow–derived monocytes and PDAC tumor cells, we showed that tumor cell–derived EVs (TC-EVs) induced monocyte differentiation toward M2-like, immunosuppressive, CD200R^+^PD-L1^+^HLA-DR^lo^ macrophages that express ALOX15B, that we identify as an independent PDAC poor-prognosis biomarker using a human PDAC metacohort. We also demonstrated that TC-EVs reprogrammed human primary PDAC CAFs, causing a fibronectin network reorganization associated with changes in extracellular matrix (ECM) composition, including alterations of WNT pathway elements such as secreted frizzled related protein-1 (SFRP1) enrichment. We also revealed that monocytes cultured on SFRP1–enriched ECM differentiated into M2-like, immunosuppressive macrophages. Last, we demonstrated that both directly and indirectly TC-EV– or SFRP1-enriched ECM–driven differentiated macrophages hindered T cell activation and subsequent antitumor activity. Our findings highlight potentially novel dual mechanisms of TC-EV–mediated crosstalk, involving ALOX15B^+^ macrophages and SFRP1^+^ CAFs, that simultaneously contribute to foster the immunosuppressive ecosystem of PDAC.

## Abstract 2

This work reveals that extracellular vesicles produced by tumor cells modulate non-tumoral cells behavior to foster the establishment of an immunosppressive ecosystem, in pancreatic cancer.

## Introduction

Pancreatic cancer (PDAC) is a devastating and fatal cancer, expected to be the third-leading cause of cancer-related deaths by 2030 in developed countries (1). These dismal characteristics are largely attributed to late diagnosis mostly established at advanced stages coupled with a lack of efficient therapies, mainly focused on targeting tumor cells. The only curative therapy for PDAC is surgical resection, for which merely 20% of patients are eligible. The majority of patients are consequently subjected to systemic chemotherapies, with rapidly acquired resistance, or palliative care (2). The recent breakthrough of immunotherapies, aiming to prime and reactivate the patient’s natural defenses against the tumor, primarily through immune checkpoint inhibitors (ICIs), has revolutionized cancer care in many solid cancers such as melanoma, non–small cell lung cancer, and colorectal cancer, among others. Unfortunately, the remarkable efficacy of these therapies has not translated to PDAC (3). Thus, understanding the immune environment of PDAC and developing new therapeutic avenues to improve PDAC patients’ fate and care are critical.

Indeed, PDAC is distinct from other solid tumors due to the presence of a highly dense, prominent, fibrotic, and immunosuppressive tumor microenvironment (TME), often accounting for the majority of the tumor bulk. This heterogeneous TME is predominated by various subtypes of constitutively activated fibroblasts, known as cancer-associated fibroblasts (CAFs), responsible for the deposition of the dense extracellular matrix (ECM) that acts as a barrier to diffusion of therapies and to the infiltration of antitumoral immune cells (4, 5). Tumor-associated macrophages (TAMs), highly immunosuppressive, antiinflammatory innate immune cells, represent the second main cellular component of PDAC TME (6). The immunosuppressive ecosystem orchestrated by the components of the TME results in exclusion or inactivity of antitumoral immune cells, such as T cells, and protects tumor cells from immune attack through antigen presentation deficiency and induction of immune tolerance. Thus, PDAC is notoriously categorized as an “immune cold” cancer type, devoid of active cytotoxic immune cells, which in part may explain the failure of immunotherapies (3, 7).

Interactions between the tumor compartment and the TME are a pronounced subject of interest in tumor biology, as we now recognize that the extensive and dynamic crosstalk between these two compartments can modulate, among others, tumor progression and antitumor immunity. Signals derived from tumor cells were found to activate resident pancreatic stellate cells to CAFs, leading to ECM remodeling and sustained CAF reprogramming to create feedback loops facilitating tumor progression (8–10). Tumor-TME communication has also been reported to recruit and maintain immunosuppressive myeloid cells, and tolerance-inducing Tregs, hindering therapeutic responses (11, 12). Cell-cell communication may occur through direct contact and secretion of soluble factors, as well as of extracellular vesicles (EVs). EVs are nanometer- to micrometer-sized cellular representations, secreted by all cell types, diffusing into tissues and bodily fluids, and capable of traveling away from their cells of origin (13). EVs, recognized as prominent mediators of intercellular communication, contain a large variety of cargo, including lipids, nucleic acids, and proteins, and are found to be secreted in high quantities by tumor cells. Particularly, PDAC tumor cell–derived EVs (TC-EVs) were found to regulate hepatic premetastatic niche formation, induce angiogenesis in hypoxic conditions, inhibit NK cell function, and confer chemoresistance, together contributing to tumor progression and metastasis (14–18).

Despite the fact that TC-EVs are highly heterogeneous and contain many layers of information, their role in the dynamic and concomitant modulation of the PDAC TME remains elusive. Accordingly, we sought to investigate the phenotypic and functional effects of PDAC TC-EVs on the establishment of a protumoral and immunosuppressive microenvironment by examining their impact on 2 key components of the TME: monocytes, the primary precursors of TAMs, and CAFs. We modeled the proximal impact of TC-EVs on macrophage trafficking by studying the differentiation of bone marrow–derived monocytes. In parallel, we investigated the impact of TC-EVs on CAF-derived ECM and revealed the consequent influence of this modified ECM on monocytes’ differentiation, highlighting a distal function of TC-EVs to foster an immunosuppressive TME. Through this study, we demonstrated that TC-EVs mediate a complex cross-communication between TME cell types. TC-EVs directly differentiate infiltrating monocytes to TAM-like, immunosuppressive macrophages, and this phenotype is mirrored with monocytes cultured on ECM produced by TC-EV–treated CAFs, providing 2 avenues of TC-EV–mediated immunosuppression, as both resulted in T cell inactivation evidenced by their reduced proliferation, expression of activation markers, and cytotoxic potential against tumor cells. Overall, our study highlights potentially novel dual mechanisms of TC-EV–mediated immunosuppression in the PDAC TME, highlighting markers of immunosuppressive CAF-ECM as well as of TC-EV–differentiated macrophages that could be potential combinatory targets or biomarkers to improve therapeutic outcomes in PDAC.

## Results

### PDAC TC-EVs drive direct healthy monocyte differentiation into M2-like macrophages.

**Figure 1:**
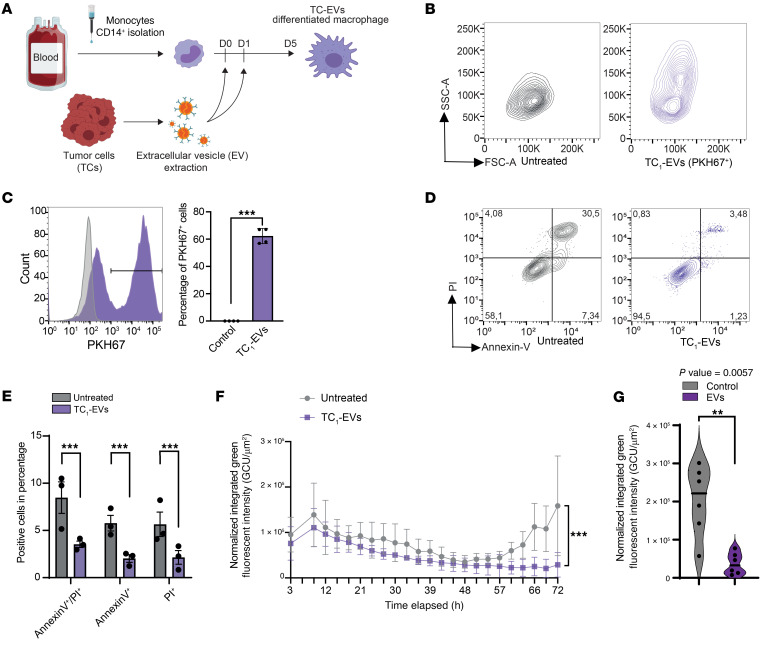
PDAC TC-EVs are efficiently internalized by monocytes and confer increased viability. (**A**) Schematic depiction of experimental protocol: healthy blood donor buffy coats were used to extract peripheral blood mononuclear cells (PBMCs), and monocytes were isolated using CD14^+^ magnetic beads. TC-EVs were extracted from TC- (TC_1_) conditioned medium, and monocytes were treated for 2 consecutive days and incubated for a total of 5 days before analysis. (**B**) Representative flow cytometry graphs of forward and side scatter of untreated and TC_1_-EV–treated monocytes 24 hours following treatment (*N* = 4). (**C**) Representative histogram of monocyte internalization of PKH67-GFP–labeled TC_1_-EVs by untreated and treated monocytes (left), 24 hours following treatments. Percentage of total cell population with internalized PKH67-GFP–labeled EVs between untreated and TC_1_-EV–treated monocytes (right), 24 hours following treatments (*N* = 4). (**D**) Representative flow cytometry plots of annexin V and propidium iodide (PI) staining of TC_1_-EV–treated monocytes following 96 hours of treatment (*N* = 3). (**E**) Percentage of total monocytes expressing annexin V, expressing PI, or double-positive for annexin V/PI in untreated and TC_1_-EV–treated monocytes (*N* = 3). (**F**) Normalized fluorescent green integrated intensity (GCU/μm^2^) of monocytes marked with dead-cell marking agent, Cytotox Green (Incucyte), in untreated and TC_1_-EV–treated monocytes over a 72-hour period analyzed by live-cell imaging (*N* = 3). (**G**) Normalized fluorescent green integrated intensity (GCU/μm^2^) of Cytotox Green in untreated or TC_1_-EV–treated monocytes at the 72-hour time point (*N* = 3). Statistical analyses were performed using paired 2-tailed Student’s *t* tests. Significant differences in expression with *P* values < 0.001 *** and < 0.01 ** are indicated.

The direct impact of TC-EVs on freshly isolated healthy monocytes’ differentiation and resulting phenotype was investigated 5 days following treatment as depicted in Figure 1A. First, cell-derived EVs were isolated by combining differential ultracentrifugation and size-exclusion chromatography (Supplemental Figure 1A; supplemental material available online with this article; https://doi.org/10.1172/JCI197733DS1); their morphologies and particle sizes/concentrations were investigated by nanoparticle tracking analysis (Supplemental Figure 1, B–D) and electron microscopy (Supplemental Figure 1E). Moreover, expression of common EV markers was assessed by Western blot (Supplemental Figure 1F). Uptake of TC_1_-released (PANC-1–released) EVs (TC_1_-EVs) by monocytes resulted in increased granularity as evidenced by higher side scatter (Figure 1B). Internalization of TC_1_-EVs by monocytes was confirmed using general lipid membrane green labeling dye PKH67-labeled TC_1_-EVs incubated with monocytes for 24 hours followed by flow cytometry analysis. On average, 60% of the total cell population had internalized EVs following 24 hours of incubation (Figure 1C). Cell viability of monocytes treated with TC_1_-EVs was assessed by annexin V/propidium iodide (PI) staining (Figure 1, D and E, and Supplemental Figure 1G) at 96 hours after TC_1_-EVs’ addition and by Incucyte live-cell imaging using Cytox Green dead-cell marking agent uptake at 72 hours after TC_1_-EVs’ addition (Figure 1, F and G). Each technical approach revealed an improvement of cell viability in TC_1_-EV–treated monocytes.

**Figure 2:**
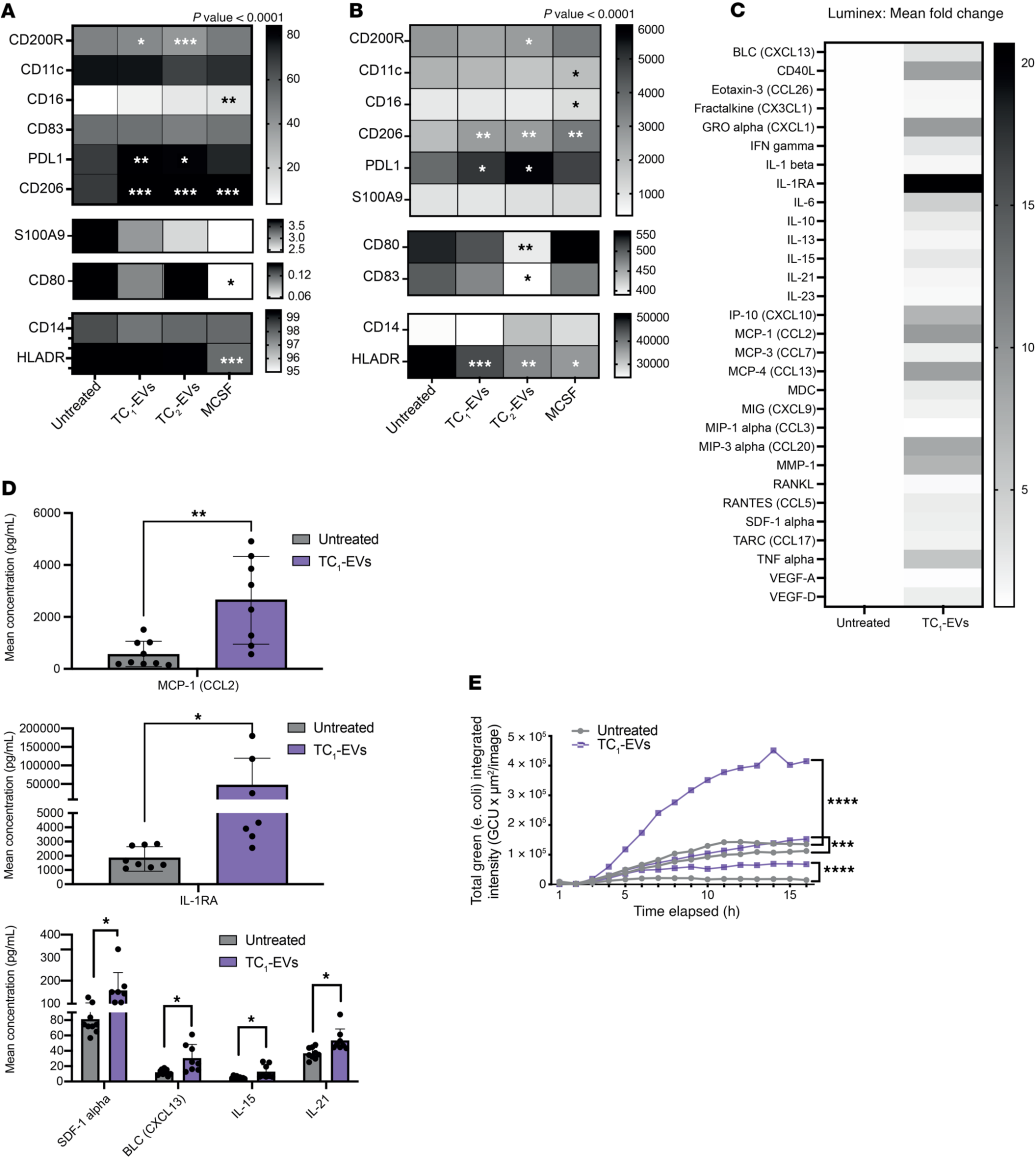
PDAC TC-EVs differentiate healthy monocytes to M2-like, immunosuppressive macrophages. (**A**) Heatmaps depicting multiparametric flow cytometry analysis of percentage of total cell populations expressing markers for monocytes/macrophages, M1 macrophages, M2 macrophages, and myeloid-derived suppressor cells (MDSCs) between untreated, TC_1_- and TC_2_-EV–differentiated macrophages, and M-CSF– differentiated (40 ng/mL) macrophages following the experimental protocol in Figure 1A (*N* = 9 to 22). (**B**) Heatmap of mean fluorescence intensity (MFI) of the markers shown in **A** (*N* = 9 to 22). (**C**) Multiplex ELISA (Luminex) analysis of 30 cytokines and chemokines secreted by untreated and TC_1_-EV–differentiated macrophages (*N* = 3). (**D**) Significantly upregulated cytokines and chemokines secreted by untreated and TC_1_-EV–differentiated macrophages (*N* = 3). (**E**) Phagocytosis of fluorescent green *E*. *coli* bioparticles by untreated and TC_1_-EV–differentiated macrophages from 3 healthy donor-derived monocytes analyzed by live-cell imaging over 16 hours (*N* = 3). Statistical analyses were performed using mixed effects analysis with uncorrected Fisher’s least significant difference (LSD) for multiple comparisons test (**A** and **B**) and paired 2-tailed Student’s *t* tests (**D** and **E**). Significant differences with *P* values < 0.0001 ****, < 0.001 ***, < 0.01 **, and < 0.05 * are indicated.

Then, we investigated the differentiation of freshly isolated healthy blood donor-derived monocytes following 5 days’ treatment with various stimuli, including PANC-1–released EVs (TC_1_-EVs) and MiaPaCa-2–released EVs (TC_2_-EVs) and nontumoral human pancreatic epithelial ductal cell–released (HPDE-released) EVs (HPDE-EVs), their respective supernatant (SN) depleted of EVs, as well as DMEM and keratinocyte serum free medium (K-SFM) derived EVs and their respective SN depleted of EVs, and M-CSF as a positive control following the experimental procedure described in Figure 1A. Resulting differentiated macrophages were analyzed by flow cytometry for the percentage of cells of the total population expressing monocyte, macrophage, and antigen-presenting cell markers (Figure 2A, Supplemental Figure 2A, and Supplemental Figure 3, A and C), and their mean fluorescence intensities (MFIs; Figure 2B, Supplemental Figure 2B, and Supplemental Figure 3, B and D). Compared with the untreated condition, treatments with TC_1_- or TC_2_-EVs resulted in a markedly increased percentage of total cell populations expressing M2 macrophage markers CD206/CD200R and immune checkpoint ligand programmed cell death ligand 1 (PD-L1) (Figure 2A and Supplemental Figure 2A) while MFI of HLA-DR was reduced by TC_1_- and TC_2_-EVs (Figure 2B and Supplemental Figure 2B). Interestingly, while some of those modified expression patterns (either in percentage or MFI) were also observed using control conditions involving conditioned medium depleted of EVs, confirming secreted elements from pancreatic tumor cells induced monocytes’ differentiation, the modified expression patterns of CD200R and PD-L1 were specific to TC_1_- or TC_2_-EVs (Supplemental Figure 3, A–D). Together, those data reveal that pancreatic TC-EV–treated monocytes differentiated toward a CD200R^+^PD-L1^+^HLA-DR^lo^ macrophage phenotype.

The secretory profile of monocytes treated with TC-EVs was analyzed by multiplex ELISA (Luminex) of 32 relevant chemokines and cytokines in the conditioned medium of control (untreated) and TC_1_-EV–differentiated macrophages. Globally, an overall increase in chemokine and cytokine production was observed (Figure 2C). Specifically, we could detect a marked increase of M2 macrophage–related chemokines/cytokines such as monocyte chemoattractive protein-1 (MCP-1)/CCL2, interleukin-1 receptor antagonist (IL-1RA), stromal cell-derived factor-1α (SDF-1α), B lymphocyte chemoattractant (BLC)/CXCL13, IL-15, and IL-21 in TC_1_-EV–differentiated macrophage–conditioned medium (Figure 2D). At the physiological level, one of the functional characteristics of M2 macrophages is phagocytosis. Thus, the phagocytic capacity of untreated and TC_1_-EV–treated macrophages was investigated by their incubation with green fluorescent *E*. *coli* bioparticles following their differentiation period. Although donor-based variability in amplitude of phagocytosis was observed, TC_1_-EV–differentiated macrophages showed a substantial increase in phagocytosis compared with untreated macrophages over a 16-hour period (Figure 2E and Supplemental Figure 2C). Together, our results reveal that PDAC TC-EVs lead monocytes’ differentiation toward physiologically active, M2-like macrophages.

### PDAC TC-EV–differentiated macrophages reduce T cell activation and cytotoxic activity toward tumor cells.

**Figure 3:**
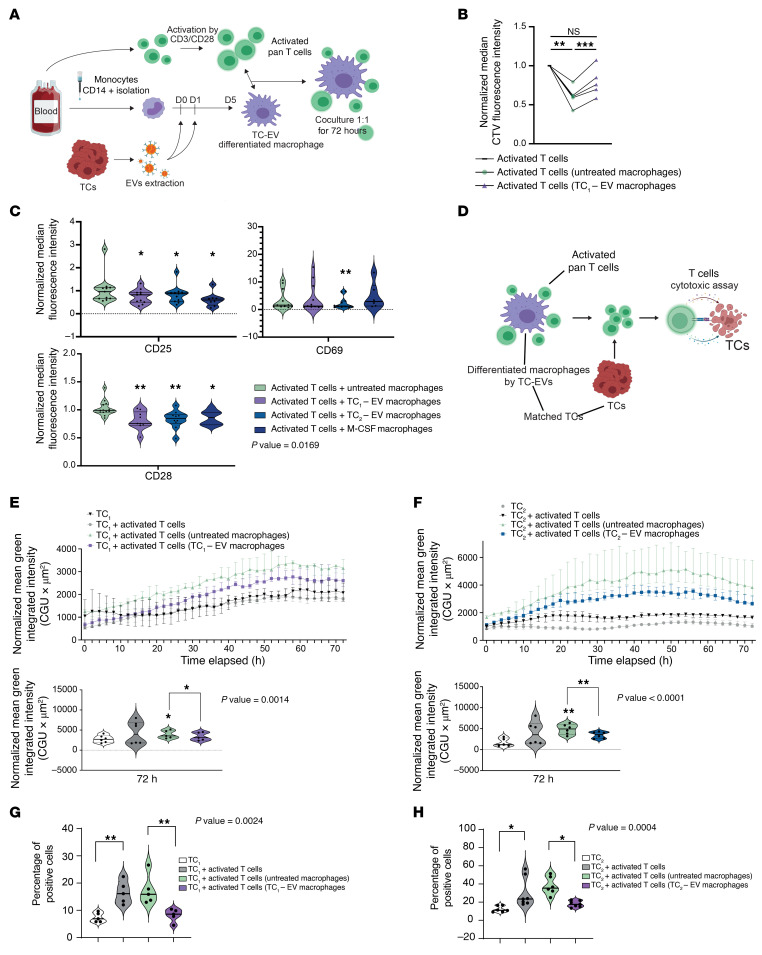
PDAC TC-EV–differentiated macrophages suppress T cell proliferation, activation, and cytotoxic activity against tumor cells. (**A**) Schematic depiction of experimental protocol. Untreated or TC-EV–differentiated macrophages were placed in coculture at a 1:1 ratio with activated T cells for 72 hours. (**B**) Proliferation assay of T cells in coculture with macrophages: fold-change of MFI of CellTrace Violet expression in T cells in coculture with untreated or TC_1_-EV–differentiated macrophages, normalized to MFI of monoculture T cells (*N* = 5). (**C**) Normalized MFI of T cell activation markers (CD25 and CD69) and costimulatory marker (CD28) in T cells cocultured with untreated, TC_1_-EV–treated, TC_2_-EV–treated, and M-CSF–treated macrophages, analyzed by flow cytometry (*N* = 4). (**D**) Schematic depiction of tumor cell killing/T cell cytotoxicity assay where T cells from macrophage cocultures were isolated and placed in coculture with TCs at a 5:1 ratio. (**E**) Caspase-3/7 activity of TC_1_ (*N* = 5) and (**F**) TC_2_ (*N* = 6) following coculture with monoculture activated T cells, cocultured T cells with untreated macrophages, and TC_1_- or TC_2_-EV–differentiated macrophages analyzed by live-cell imaging over 72 hours (*N* = 5) (top). Graphs of caspase-3/7 activity of both TCs in above conditions at 72 hours (bottom). (**G** and **H**) Total percentage of TC_1_ (**G**, *N* = 5) and TC_2_ (**H**, *N* = 6 or 7) cells positive for annexin V or propidium iodide (PI) and double positive for annexin V/PI following cocultures with above-described T cell conditions analyzed by flow cytometry. Statistical analyses were performed using paired 2-tailed Student’s *t* tests (**B**), mixed-effect analysis with uncorrected Fisher’s LSD for multiple comparisons test (**C**), 1-way nonparametric ANOVA Kruskal-Wallis test, and Dunn’s multiple comparisons tests (**E**–**H**). Significant differences with *P* values < 0.01 ** and < 0.05 * are indicated.

Since TC-EV–differentiated macrophages demonstrated an M2-like phenotype, which is known to correlate with immunosuppressive abilities (19), we evaluated their functional impact on T cells’ activity/activation. Following the previous protocol, the differentiated macrophages were cocultivated for 72 hours with same-donor activated T cells at a 1:1 ratio as depicted in Figure 3A. First, we analyzed their proliferative activity using the CellTrace Violet assay and observed that while activated T cells from coculture with untreated macrophages showed reduced normalized mean CFSE fluorescence intensity, reflecting an increase in T cells’ proliferation and thus their increased activation, activated T cells from the same donors in coculture with TC_1_-EV–differentiated macrophages showed a decreased proliferation as shown by higher normalized mean CFSE fluorescence intensity (Figure 3B and Supplemental Figure 4A). In agreement with this T cell proliferation, we showed that expression of T cell activation markers CD25 and CD69, and costimulatory factor CD28, was decreased in activated T cells cocultured with TC-EV–differentiated macrophages compared with those cocultured with untreated macrophages (Figure 3C). Finally, to analyze whether these T cells, with reduced proliferation/expression of activation markers, had a modulated cytotoxic potential, we implemented the previous protocol (Figure 3A) with final coculture with either TC_1_ or TC_2_, depending on the TC-EVs used to induce monocyte differentiation, to evaluate their tumor cell–killing ability (Figure 3D). Through live imaging of cocultures and incubation with Incucyte caspase-3/7 reagent, both TC_1_ and TC_2_ revealed reduced caspase-3/7 staining when cultivated with T cells from their respective TC-EV–differentiated macrophage cocultures compared with cocultures with untreated macrophages (Figure 3, E and F). Similarly, cumulative percentages of cells positive for annexin V, PI^+^ cells, and double-positive annexin V^+^/PI^+^ TCs were reduced when cocultivated with T cells from their respective TC-EV–differentiated macrophage cocultures compared with T cells from untreated macrophage cocultures (Figure 3, G and H, and Supplemental Figure 4B), indicative of reduced TC death and T cell cytotoxic potential. Overall, those results demonstrate that TC-EV–differentiated macrophages drive a reduced proliferation, activation, and cytotoxic activity of T cells.

### PDAC TC-EV–differentiated macrophages highlight a distinct protein signature.

**Figure 4:**
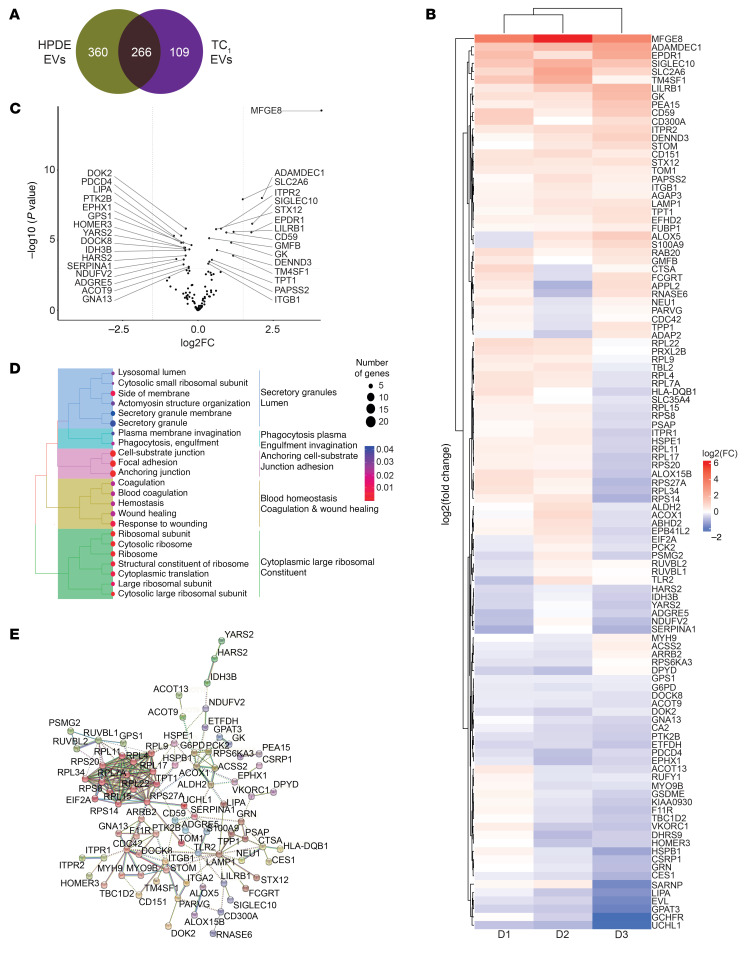
PDAC TC-EV–differentiated macrophages have a proteomic profile enriched with ribosome proteins and protein synthesis pathways. (**A**) Venn diagram comparing the number of differentially expressed proteins in TC_1_-EV–differentiated macrophages with HPDE-EV–differentiated macrophages (*N* = 3). (**B**) Heatmap of significantly differentially expressed proteins in 3 healthy blood donor-derived monocytes differentiated by TC_1_ EVs. (**C**) Volcano plot of significantly up- and downregulated proteins in TC_1_-EVs differentiated macrophages compared with untreated macrophages. (**D**) Overrepresentation analysis (ORA) tree plot of differentially expressed proteins in TC_1_-EV–differentiated macrophages. (**E**) STRING protein interaction network of differentially expressed proteins in TC_1_-EV–differentiated macrophages.

We then investigated the proteomic profile of TC-EV–differentiated macrophages to establish a TC-EV–correlated specific signature of these macrophages. We prepared 3 biologically independent experiments of TC_1_, TC_2_, and nontumoral pancreatic ductal cell (HPDE) EV–differentiated macrophages (from 3 independent blood donors), for proteomic analysis by mass spectrometry (MS), following the same treatment design as in Figure 1A. As HPDE-EVs were not able to induce the M2-like differentiation of monocytes observed following TC-EV treatments with the phenotype of CD200R^+^PD-L1^+^HLA-DR^lo^ (Figure 2, A and B, and Supplemental Figure 3, A and B), we considered that significantly dysregulated proteins following HPDE-EV treatments (Figure 4A and Supplemental Figure 5A) could not be part of our specific signature of interest, or responsible for monocytes’ differentiation, and consequently, we removed those proteins from our following investigations. The nonspecific TC-EVs’ impact on monocytes’ protein content was accounted for by not considering differentially expressed proteins in monocytes treated by HPDE-EVs (Figure 4A and Supplemental Figure 5A).

To account for donor variability, proteins that were differentially expressed in 2 out of 3 donors were considered. Thus, we were able to highlight 109 and 54 differentially expressed proteins in macrophages specifically treated with TC_1_- and TC_2_-EVs, respectively (Figure 4, A–C, and Supplemental Figure 5, A–C). Pathway enrichment analysis revealed enrichment of proteins primarily in cytoplasmic large ribosome constituent, blood coagulation/healing, anchoring cell-substrate junction adhesion, phagocytosis, and secretory actomyosin granules in TC_1_-EV–differentiated macrophages (Figure 4D) and in cytoplasmic large ribosomal subunit, anchoring cell-substrate focal junction, initiation factor granule, and polysome ribosomes in TC_2_-EV–differentiated macrophages (Supplemental Figure 5D). The use of STRING analysis to denote protein interactions, with Markov-cluster algorithm clustering, revealing 22 and 10 clusters in TC_1_- and TC_2_-EV–differentiated macrophages, respectively (Figure 4E and Supplemental Figure 5E), and the enriched pathways’ interactions were summarized in a radial plot for the TC_1_- and TC_2_-EV–differentiated macrophages, respectively (Supplemental Figure 5, F and G). Interestingly, only 12 commonly differentially expressed proteins were found between TC_1_- and TC_2_-EV–differentiated macrophages, representing a specific protein signature of macrophages’ differentiation driven by TC-EVs (Table 1)**.**

### ALOX15B from the signature of PDAC TC-EV–differentiated macrophages correlates with poor prognosis.

**Figure 5:**
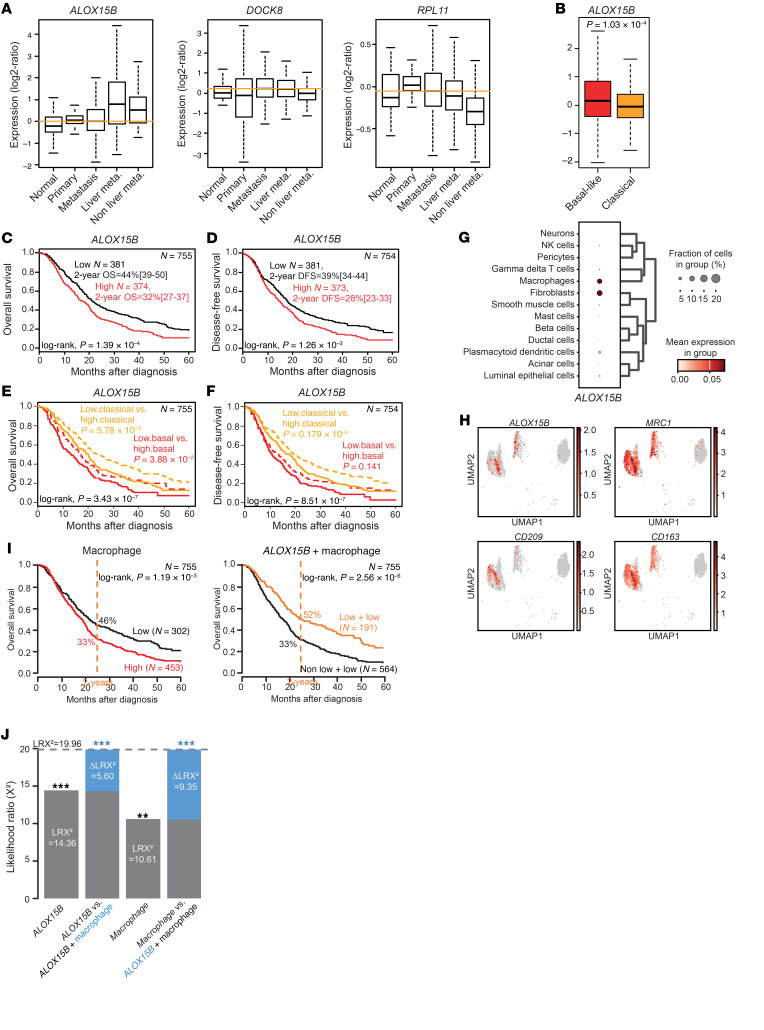
*ALOX15B* expression correlates with poor patient outcomes and associates with M2-like macrophages in PDAC. (**A**) Box plot of *ALOX15B*, *DOCK8*, and *RPL11* mRNA expression (log_2_) as continuous values in PDAC primary (*N* = 938), PDAC metastasis (*N* = 152), and normal pancreatic samples (*N* = 158). Orange line represents median expression level across cohort. Boxes represent IQR with median line; whiskers extend to farthest data points within standard 1.5× IQR outlier threshold. *ALOX15B*, arachidonate 15-lipoxygenase type B. (**B**) Box plot of *ALOX15B* mRNA expression (log_2_) in PDAC primary samples per molecular subtypes of Moffitt et al. *P* value is for 2-tailed Student’s *t* test. (**C** and **D**) Kaplan-Meier curve of OS (**C**) and DFS (**D**) in PDAC according to *ALOX15B* expression, high or low. (**E**) Kaplan-Meier curve of OS in PDAC according to *ALOX15B* expression, high or low, and basal-like or classical Moffitt PDAC subtype. Low.Basal_Like *N* = 110, 2-Year OS=32%[24–43], high.Basal_like *N* = 152, 2-Year OS=25%[19–34]. Low.Classical *N* = 271, 2-Year OS=50%[44–57], high.Classical *N* = 222, 2-Year OS=36%[30–44]. (**F**) Kaplan-Meier curve of DFS in PDAC according to *ALOX15B* expression, high or low, and basal-like or classical Moffitt PDAC subtype. Low.Basal_Like *N* = 110, 2-Year OS=27%[19–37], high.Basal_like *N* = 151, 2-Year OS=20%[14–28]. Low.Classical *N* = 271, 2-Year OS=44%[38–50], high.Classical *N* = 222, 2-Year OS=33%[27–40]. (**G**) Dot plot results of *ALOX15B* in cell types from PDAC (GSE197177). (**H**) Uniform manifold approximation and projection (UMAP) of leiden clusters and localization and expression of *ALOX15B* and M2 markers (*CD206*, *CD209*, *CD163*) from PDAC (GSE197177). (**I**) Kaplan-Meier curve of OS in PDAC according to enrichment of macrophage scores, high or low (left), and according to *ALOX15B* expression combined with macrophage score, low *ALOX15B*-low macrophage score expression, and non-low-*ALOX15B* or non-low macrophage score expression (right). (**J**) Comparison of prognostic information for OS of *ALOX15B* expression alone, macrophage score alone, and combination of *ALOX15B* expression and macrophage score in human PDAC using likelihood ratio analysis.

We interrogated the clinical relevance of the expression of each gene coding for those 12 proteins by correlating their relative mRNA expression with clinical characteristics in a large PDAC cohort (Table 2). A total of 1,248 samples were analyzed, including 158 normal pancreas, 938 primary tumors, and 152 metastasis samples. The expression pattern of the 12 members of the signature revealed significant modulations in correlation with the origin of tumor samples (Figure 5A and Supplemental Figure 6, A–C). Among those 12 markers, we focused our attention on *Alox15b*, as its high expression correlated with the basal-like PDAC subtype, which is a poor-prognosis subtype (Figure 5B), and *Alox15b* expression was the only significant poor-prognosis marker for overall survival (OS) and disease-free survival (DFS) in the whole cohort of primary tumors (Figure 5, C and D, and Supplemental Figure 6, D and E). The patients with high expression showed 32% 5-year OS (95%CI 27–37) and 28% 5-year DFS (95%CI 23–33) versus 44% (95%CI 39–50) and 39% 5-year DFS (95%CI 34–44), respectively, for those with low expression. Interestingly, high *Alox15b* expression retained its poor-prognosis value in multivariate analysis for both DFS and OS (data not shown), suggesting independent prognostic value from Moffitt’s molecular subtypes notably. High *Alox15b* expression was associated with lower DFS and OS in both Moffitt’s subtypes (Figure 5, E and F). We further validated the expression of *Alox15b* in macrophages using our clinical cohort of PDAC bulk transcriptomes (Supplemental Figure 6F), as well as single-cell RNA-sequencing public datasets (Figure 5G), and even more specifically in M2 macrophages using some major M2 markers, such as *CD206*, *CD163*, or *CD209* (Figure 5H and Table 3), and M1 markers, such as *CD83* and *CD74* (Supplemental Figure 6G and Table 3). Finally, we compared the prognostic information for OS provided by *ALOX15B* expression alone, by the Bindea’s macrophage score alone, and the combination of both. The macrophage score displayed prognostic value with shorter OS associated with higher score (Figure 5I). Given this observation and the prognostic value of *ALOX15B* expression, and given the correlation between both variables, we searched for an eventual prognostic value of a model combining both of them. As shown in Figure 5I, this model was prognostic, with longer OS in patients with both low *ALOX15B* expression and low macrophage score than in patients without low ALOX15B expression and low macrophage score. Interestingly, the combined model provided more prognostic information than each variable separately (Figure 5J and Supplemental Figure 6H), and each variable significantly contributed to enhance the prognostic value of the other one (*P* < 0.05 for the 2 Δlikelihood ratio χ^2^). Together these data demonstrate that among the specific protein signature of M2-like macrophages’ differentiation driven by TC-EVs, the expression of *Alox15b* correlates with poor prognosis and more aggressive tumors in PDAC, independently from classical prognostic features.

### PDAC TC-EVs influence primary CAFs’ structuration of fibronectin fibers.

**Figure 6:**
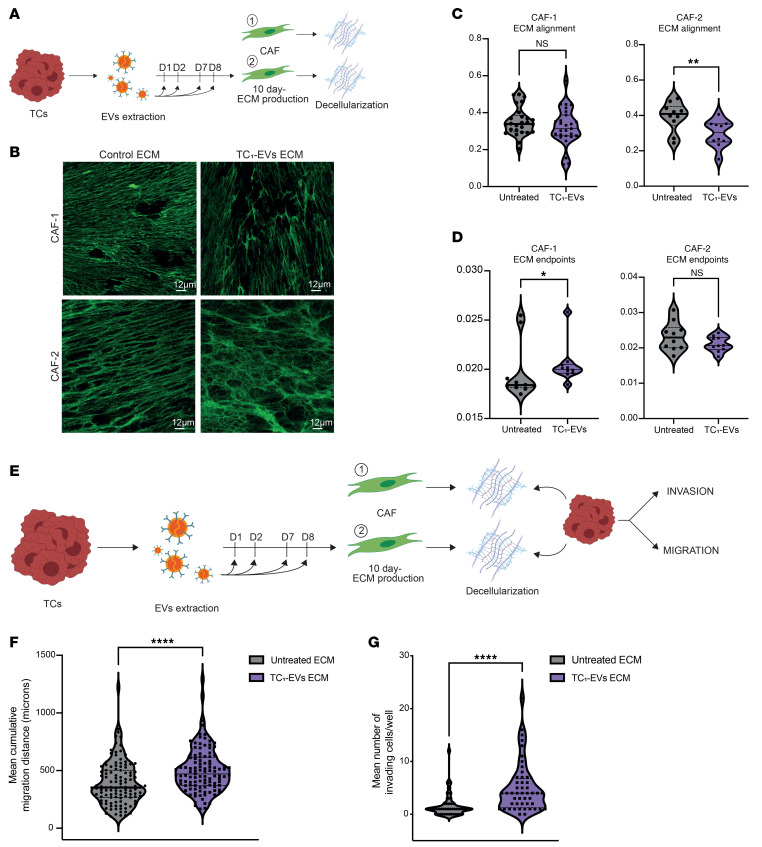
PDAC TC-EVs modify structural features of CAF-derived ECM and promote TCs’ aggressiveness. (**A**) Schematic depiction of experimental protocol: Primary CAFs were cultured for a period of 8 days during which they were treated 4 times, for 2 consecutive days, with TC-EVs. Following the culture period, ECM was decellularized to remove CAFs and used for immunofluorescence staining or other functional studies. (**B**) Representative confocal microscopy images of fibronectin fibers (green) produced by untreated or TC_1_-EV–treated primary CAFs derived from 2 patients. Scale bars: 12 μm. (**C**) Quantification of fibronectin fiber alignment by TWOMBLI of 2 primary CAF-derived ECM (*N* = 3). (**D**) Quantification of fibronectin fiber endpoints by TWOMBLI of 2 primary CAF-derived ECM (*N* = 3). (**C** and **D**) The *y* axis shows value for alignment and endpoints. TWOMBLI generates a raw values matrix for comparison of ECM patterns. (**E**) Schematic depiction of ECM production by CAFs as in **A** followed by plating of tumor cells for migration and invasion assays. (**F**) Migratory capacity, in mean migration distance (microns), of TC_1_ on ECM produced by untreated or TC_1_-EV–treated CAFs analyzed by time-lapse microscopy over 12 hours (*N* = 3). (**G**) Invasive capacity, in mean number of invading cells, of TC_1_ on ECM produced by untreated or TC_1_-EV–treated CAFs through a Boyden chamber (8-micron pores) assay at 24 hours (*N* = 3). Statistical analyses were performed using unpaired 2-tailed Student’s *t* tests. Significant differences with *P* values < 0.0001 ****, < 0.01 **, and < 0.05 * are indicated.

We then investigated the indirect impact of TC-EVs on freshly isolated healthy monocytes’ differentiation. As PDAC-associated immune cells are evolving within a dense ECM network, we hypothesized that TC-EVs could influence monocytes’ differentiation through a modulation of the CAF-derived ECM. We first evaluated the impact of TC-EVs on CAF-produced ECM using 2 PDAC patients’ primary CAFs, treated with TC_1_- or TC_2_-EVs during an 8-day incubation period required for cell-derived ECM production (Figure 6A). At the end of the 8-day period, ECM was decellularized and analyzed by immunofluorescence for fibronectin fiber structural characterization. Confocal microscopy images show less uniform directionality as well as less dense and more porous fibronectin fibers in the TC-EV–treated CAF conditions compared with controls (Figure 6B). Structural features of these fibronectin fibers were quantified using the ImageJ plugin TWOMBLI (The Workflow Of Matrix BioLogy Informatic), a pipeline allowing for the quantification of various features (matrix fiber alignment, endpoints). Interestingly, quantification of fiber alignment showed lower alignment of fibronectin fibers in the TC-EV–treated condition for 1 type of primary CAF (Figure 6C) while the number of normalized fiber endpoints per unit length was increased in TC-EV–treated condition for the other primary CAF (Figure 6D). Quantification of other TWOMBLI parameters, such as lacunarity, fiber length, branchpoints, curvature, hyphal growth unit, and fractal dimension, were also significantly different between both primary CAF-derived ECM but always dependent on TC-EV treatment (Supplemental Figure 7, A and B). Interestingly, ECM produced by CAF treated with HPDE-EVs does not recapitulate structural features’ modification such as matrix fiber alignment (Supplemental Figure 8, A and B). Those data reveal that, while reflecting CAFs heterogeneity among patients with PDAC, TC-EVs are able to specifically modulate CAF-derived fibronectin structuration.

### ECM produced by PDAC TC-EV–treated CAFs enhances PDAC tumor cells’ aggressiveness.

To assess the biological relevance, in correlation with PDAC aggressiveness, of TC-EV–treated CAF–derived ECM, TC_1_ and TC_2_ were plated on untreated or their corresponding TC_1_/TC_2_-EV–treated CAF–derived ECM. Then we evaluated their migratory and invasion abilities (Figure 6E). We observed that TC_1_ and TC_2_ cells showed improved migratory capacities, through time-lapse microscopy over a 12-hour period (Figure 6F and Supplemental Figure 8C). Specifically, total migration distance of each tumor cell type was increased where speed and total displacement were not significantly impacted by plating on EV-treated CAF-ECM (Supplemental Figure 7, C and D). We further observed that both TC_1_ and TC_2_ cells invaded more through their respective EV-treated CAF–derived ECM than control ECM (Figure 6G and Supplemental Figure 7, E–H). Thus, TC-EV–treated CAFs produce a protumoral ECM favoring pancreatic tumor cells’ aggressiveness.

### PDAC TC-EV–treated CAFs produce ECM with increased expression of Wnt pathway proteins SFRP1 and Wnt5a.

**Figure 7:**
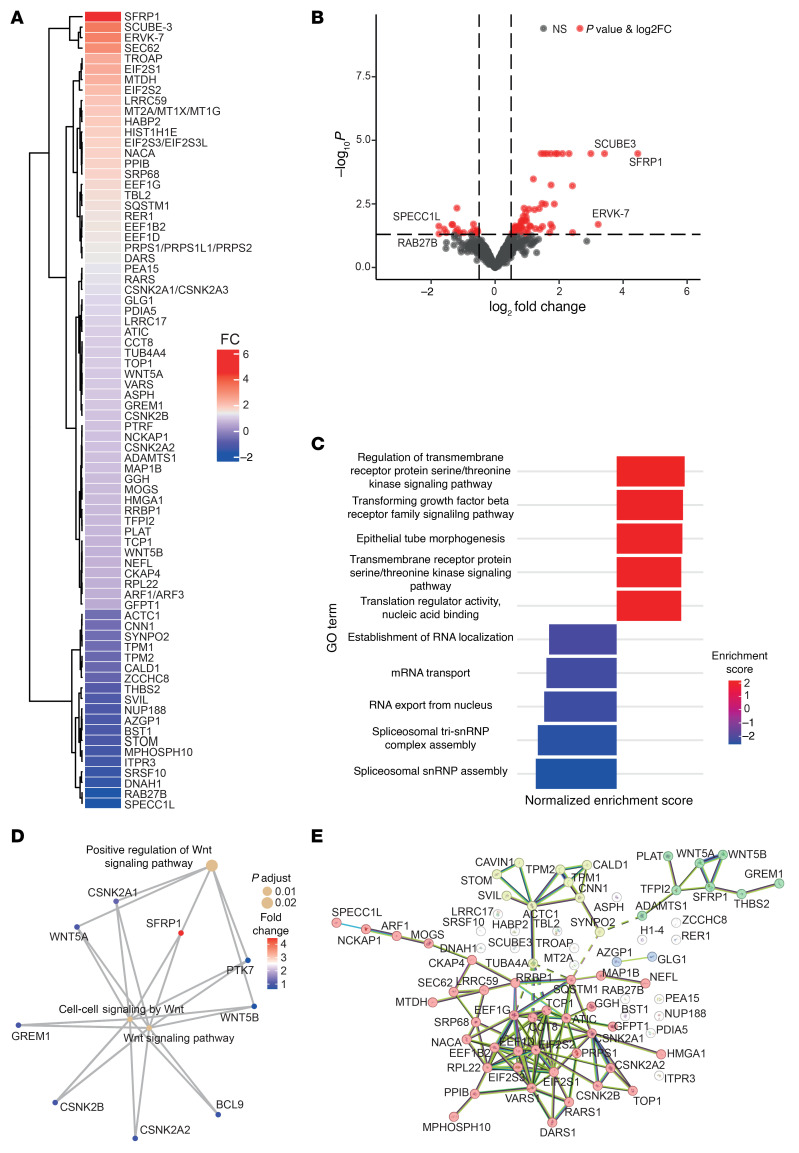
PDAC TC-EV–treated CAFs produce ECM enriched in Wnt pathway proteins. (**A**) Mean log_2_ fold-changes of protein expression in CAF-derived ECM treated with TC_1_-EVs compared with untreated CAF-ECM (*N* = 3). SFRP1, secreted frizzled related protein 1. (**B**) Differentially expressed proteins in CAF-derived ECM treated with TC_1_-EVs compared with untreated CAF-ECM. (**C**) Gene ontology enrichment analysis depicting differentially expressed protein pathways in TC_1_-EV–treated CAF-derived ECM. Terms associated with the proteins from the heatmap were selected and the top 10, including the 5 most upregulated and 5 most downregulated terms, identified. (**D**) Network plot showing the relationship of SFRP1 and other genes in the Wnt signaling pathways and their relative fold-changes between untreated and TC_1_-EV–treated CAF-ECM. (**E**) Protein network analysis of TC_1_-EV–treated CAF-ECM.

To investigate how TC-EVs may modify the composition of the ECM deposited by CAFs, and whether the potentially modified composition contributes to the increased aggressiveness of TCs, we performed proteomics evaluation of CAF-ECM (matrisome), using control ECM or ECM treated with TC_1_-EVs by liquid chromatography-tandem mass spectrometry (LC-MS/MS). Heatmap and volcano plots show 76 differentially expressed proteins in TC_1_-EV–treated CAF–derived ECM (*P* < 0.05, log_2_ fold-change < or > 0.5) (Figure 7, A and B). Through gene enrichment analyses, differentially expressed proteins found in the TC_1_-EV–treated CAF-ECM group were found to be primarily enriched in regulation of transmembrane receptor protein serine/threonine kinase signaling pathway and transforming growth factor-β receptor superfamily signaling pathway, among others (Figure 7C). By combining those differential analyses, we focused our attention on the Gene Ontology term Wnt signaling pathway (enrichment score of 0.705; *P* value of 0.0001), which involves the most enriched protein, SFRP1, with a log_2_ fold-change of 4.46 (Figure 7B). In line with our hypothesis, recent evidence suggests SFRP1^+^ CAFs as modulator of the immune ecosystem (20). Using Kyoto Encyclopedia of Genes and Genomes we could reveal the WNT protein cluster and highlight SFRP1 as the unique upregulated protein in TC_1_-EV–treated CAF-ECM, linking the 3 Wnt pathways present in the analysis (Figure 7D). STRING analyses confirmed such clustering with a Wnt protein cluster with SFRP1 as a core central linking protein (Figure 7E). Therefore, the ECM produced by CAFs following TC-EV treatment revealed marked modification in protein content such as WNT elements and notably an enrichment in SFRP1.

### SFRP1 in TC-EV–treated CAF–derived ECM favors monocyte-to-macrophage differentiation to reduce T cell antitumoral activity.

**Figure 8:**
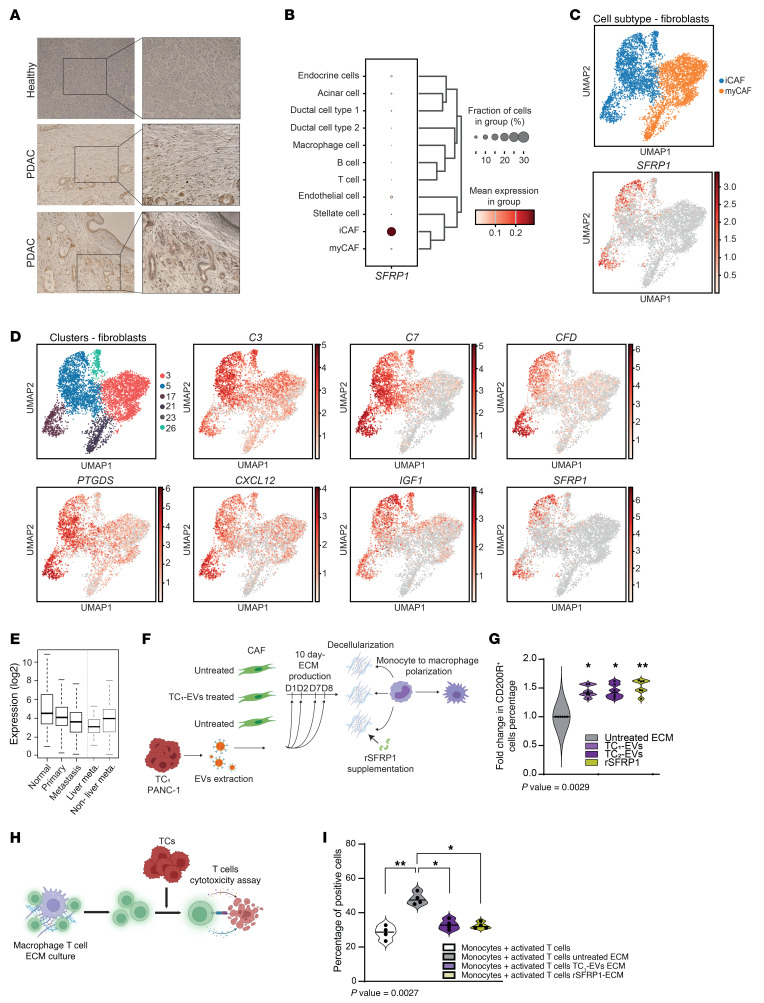
SFRP1 is expressed in a subset of inflammatory CAFs in human PDAC and suppresses T cell activity and cytotoxicity. (**A**) IHC of SFRP1 expression in human healthy pancreas and PDAC tissue microarrays. Images taken at 20× (left) and 40× (right) original magnification. (**B**) Dot plot results of *SFRP1* in cell types from PDAC (CRA001160). iCAF, inflammatory CAF; myCAF, myofibroblastic CAF. (**C**) UMAP of CAF subtypes and expression of *SFRP1*. (**D**) UMAP of leiden clusters of fibroblast cell types and expression of *SFRP1* and iCAF markers (*C3*, *C7*, *CFD*, *PTGDS*, *CXCL12*, *IGF1*) in PDAC (CRA001160). (**E**) Box plot of *SFRP1* mRNA expression (log_2_) in PDAC primary samples (*N* = 938), metastasis samples (*N* = 152), and normal pancreatic samples (*N* = 158). Whiskers extend to the farthest data points within the standard 1.5× IQR outlier threshold, and the line delineates a subanalysis focused on metastases only. (**F**) Schematic depiction of experimental protocol. Following decellularization, healthy blood donor-derived monocytes were extracted as previously described and plated on decellularized ECM without or with recombinant SFRP1 (rSFRP1). (**G**) Fold change of CD200R^+^ cell percentage on macrophages plated for 5 days on decellularized ECM derived from untreated CAFs, CAFs treated with TC_1_-EVs, TC_2_-EVs, or rSFRP1-supplemented ECM (*N* = 6, matched samples). (**H**) Schematic representation of experimental protocol. (**I**) Total percentage of cells positive for annexin V or for propidium iodide (PI) and double positive for annexin V/PI following 72 hours’ coculture with activated T cells or activated T cells cultured on decellularized ECM derived from untreated CAFs, CAFs treated with TC_1_-EVs, and rSFRP1 supplemented ECM (*N* = 4). Statistical analyses were performed using 1-way nonparametric ANOVA Kruskal-Wallis test and Dunn’s multiple comparisons tests (**G** and **I**). Significant differences with *P* values < 0.01 ** and < 0.05 * are indicated.

We validated our matrisome findings using PDAC patient samples and investigated for SFRP1 level in healthy pancreas, PDAC tissue samples, and publicly available datasets of PDAC patient cohorts. We observed that SFRP1 level was detected in PDAC samples only (Figure 8A), with a specific staining detected in nontumor cells along with its expression in the tumoral compartment (Figure 8A). CAFs being the main ECM producer in PDAC, we searched for CAF expression of SFRP1. Using single-cell RNA sequencing from publicly available datasets, our investigation revealed that *SFRP1* is indeed expressed in CAFs (Figure 8, B and C). More specifically, we could determine that *SFRP1* is expressed by a specific subset of CAFs, the iCAFs, as shown by a strong correlation with known major iCAF markers such as C3, C7, CFD, PTGDS, or CXCL12 (Figure 8D and Table 4). In parallel, we could reveal that *SFRP1* expression in CAFs does not correlate with myCAF markers, *MMP11* and periostin (Figure 8B and Supplemental Figure 9A), and does not colocalize with α-smooth muscle actin (α-SMA), a master myCAF marker (Supplemental Figure 9B). Interestingly, *SFRP1*^+^ CAFs highly correlate with *IGF1*^+^ CAFs (Figure 8D), described as an early activated state that represent the predominant iCAF described in PDAC in the current literature, and differ from *IL11*^+^ iCAFs that correlate with inflammatory monocytes’ infiltration in PDAC (21). We further assessed the clinical relevance of *SFRP1* expression in human PDAC by analyzing mRNA expression levels in the 1,248 samples of our gene expression database. *SFRP1* expression was variable according to the sample’s origin (Figure 8E). We defined high- and low-expression groups based on median *SFRP1* mRNA expression. We showed that patients with *SFRP1*-high PDAC were associated with an increased EMT Metagene score, an increased Bindea macrophage score, as well as the squamous subtype in Bailey’s classification, the quasi-mesenchymal subtype in Collisson’s classification, and the basal-like subtype on Moffitt’s classification, which represent the subtypes with worst OS (22) (Table 5).

Since both *SFRP1*^+^ CAFs and iCAFs (correlating with *SFRP1* expression) have been described as modulators of tumoral immune response (21, 23) (Supplemental Figure 9, C–E), we decided to evaluate their impact on monocytes’ differentiation as compared with the direct impact of TC-EVs on monocytic differentiation previously described (Figures 1–5). Monocytes were extracted from healthy human blood donors as previously described and plated on decellularized CAF-derived ECM, untreated, treated with TC_1_-EVs or TC_2_-EVs, or supplemented with rSFRP1 (Figure 8F). After 5 days’ culture on ECM, following immunophenotyping using flow cytometry, we observed that monocytes plated on TC_1_- or TC_2_-treated CAF–derived ECM and rSFRP1-supplemented CAF-derived ECM showed an increased frequency of positive population expressing M2 macrophage marker CD200R (Figure 8G). This evidence is in line with the positive correlation we observed between *CD163*^+^ macrophages and *IGF1*^+^ iCAFs (Supplemental Figure 9, F and G). After 5 days of monocyte culture on ECM, same donor activated T cells were added for 72 hours before being extracted for cocultures with TCs for cytotoxicity analysis using annexin V/PI staining of TCs and evaluated after 24 hours (Figure 8H). We observed that T cells from TC_1_-EV–treated CAF–derived ECM and rSFRP1-supplemented CAF-derived ECM had reduced cytotoxic potential against pancreatic tumor cells indicative of a decreased activation state of T cells (Figure 8I). Overall, our data demonstrate that TC-EVs drive CAF reprogramming to produce an SFRP1-enriched ECM that consequently impacts monocyte-to-macrophage polarization and T cell activity, fostering the development of an immunosuppressive ecosystem.

## Discussion

PDAC remains one of the most lethal malignances, distinguished by a grim prognosis with a 5-year survival rate which barely reaches 10% to 12%. Even with the emergence of more efficient chemotherapeutic regimens such as FOLFIRINOX, PDAC patients’ survival was increased by merely a few months in the last 20 years (24). While numerous solid tumors showed important progress in patient survival notably due to the rise of immunotherapies, no improvements have been reported thus far for PDAC. ICI therapies are efficient for less than 1% of patients with PDAC. This failure is imputed to the specific and prevalent nature of the intratumoral microenvironment of these tumors that is considered as highly fibrotic and immunologically “cold.” While immune cells of the TME have already been reported as key actors of immunotherapy resistance (25), the TME of PDAC is mostly infiltrated with immunosuppressive cells such as Tregs, MDSCs, and TAMs, which contribute to immune evasion and resistance to immunotherapies (26). The PDAC TME is further characterized by a deficiency in adaptive T cell immunity, with the absence of effector T cells, explaining the tumor’s disability to further respond to ICIs. Thus, understanding the immune ecosystem of PDAC and the relationship established by immune cells with other PDAC cell components is critical to optimize available immunotherapies or even further develop emerging immunotherapy strategies.

Intercellular communication between PDAC tumor cells and components of the TME is essential in regulating tumor progression by activation of CAFs and formation of the desmoplastic reaction, hindrance of immune surveillance, angiogenesis, and resistance to chemotherapies (27, 28). EVs are prominent mediators of this communication, capable of systemic circulation, diffusing at great distances from their various cells of origin while carrying simultaneously multiple cargo with various methods in transmission of their information, multiplying their communicative potential (29). These unique characteristics of EVs highlight them as intriguing actors of tumor-driven immune evasion and tumor progression yet remain largely unexplored in PDAC. Here, we demonstrated that TC-EVs are key modulators of 2 main cellular actors within the PDAC TME, namely CAFs and macrophages, in order to foster an immunosuppressive ecosystem. TC-EVs are capable (a) to directly differentiate monocytes to M2-like immunosuppressive macrophages with a unique protein expression profile with ALOX15B as associated marker and (b) to indirectly favor the formation of M2-like macrophages through the formation of a specifically SFRP1-enriched ECM produced by CAFs. Together, both paths of macrophage differentiation led to T cell inactivation and reduction of their cytotoxic potential. These findings highlight potentially novel mechanisms of monocyte-derived macrophage differentiation simultaneously at proximal and distal levels, with TC-EVs at their core, supporting immune evasion and tumor aggressiveness in PDAC.

High levels of circulating and recruited monocytes in PDAC have been found to be linked to poor prognosis, tumor progression, and immune checkpoint blockade (30–32). Monocytes are the most prominent source of TAMs compared with embryonic-origin macrophages and play a key role in antigen presentation and thus immune escape and tumor growth (33, 34). As tumor-derived EVs and monocytes both circulate systemically in blood and these EVs can be in contact with tumor-infiltrating monocytes, we hypothesized that TC-EVs can influence monocyte-to-macrophage differentiation locally or at a distance and consequently support immune evasion. Our findings confirmed the internalization of tumor-derived EVs by monocytes, which was recently found to be mediated by transmembrane protein CD44H on monocytes and resulted in activation of the STAT3 pathway (35). Interestingly, we have demonstrated increased survival and direct differentiation of healthy monocytes into alternatively activated M2/TAM-like immunosuppressive macrophages by EVs generated from 2 PDAC tumor cells, without the need for classical differentiation cytokines such as M-CSF. Classical monocytes normally circulate for 1 day in blood in steady states (36). It was previously found that EVs derived from lung, breast, and hepatocellular carcinoma cells supported monocyte survival in line with our findings (37). To be an active source of TAMs in the TME and to continually support immunosuppression, TC-EVs in PDAC may thus promote monocyte survival and subsequent infiltration and attraction into tumor tissues. Analysis of monocyte/macrophage marker expression allowed us to demonstrate variable expression of markers in TC_1_- and TC_2_-EV–treated monocytes, first denoting the heterogenous action of different tumor cell line–derived EVs on monocyte differentiation. Nonetheless, a consistent increase of well-established M2 macrophage marker, CD200R1, associated with phagocytosis and poor prognosis in PDAC (38), was observed in both TC_1_- and TC_2_-EV–treated monocytes. Immune checkpoints, against which immunotherapies are targeted, control T cell activation and responses and are largely manipulated by tumors to evade the immune response in multiple cancers. Here, we demonstrated that TC_1_- and TC_2_-EVs, but not M-CSF, were capable of inducing higher expression of the immune checkpoint ligand PD-L1, demonstrating an EV-induced immunosuppressive characteristic of macrophages in PDAC. Clinical correlation analyses of the proteomic signature from TC-EV–differentiated macrophages we highlighted allowed for the identification of arachidonate 15-lipoxygenase type II (15-LOX2), encoded by the gene *ALOX15B*, as upregulated in TC-EV–differentiated macrophages in correlation with increased metastases and poor disease-free and overall survivals. 15-LOX2, conversion of polyunsaturated fatty acids to hydroxy fatty acids, is constitutively expressed in human monocyte-derived macrophages and related to efferocytosis and ferroptosis. Expression of 15-LOX2 specifically by TAMs can result in an increase of bioactive lipid molecules in the TME and contribute to tumorigenesis as well as modify macrophage functions in relation to antitumoral immunity. IL-13–induced macrophages were associated with 15-LOX2 expression, as part of a leukotriene synthesis–related M2 signature, as well as T cell dysfunction, immune checkpoint expression, and unfavorable outcomes in glioma and had high CCL2 secretion in renal cell carcinoma (39, 40). In line with the above findings, we demonstrated the high expression of 15-LOX2 in PDAC TC-EV–differentiated macrophages, associated with CD200R^+^PD-L1^+^HLA-DR^lo^ expression, immunosuppressive cytokine and chemokine secretion, impedance of T cell activation, and poor patient outcome. Future studies investigating the mechanistic regulation of 15-LOX2 increase in macrophages, following TC-EVs, could shed light on a potential new target to control macrophage-to-lymphocyte interactions resulting in immunosuppression.

Under the influence of tumor-derived signals, TAMs secrete cytokines and chemokines promoting tumor growth and metastases and mediating immune suppression (41–43). Among the secreted factors analyzed, TC-EV–differentiated macrophages showed the highest expression of MCP-1/CCL2 and IL-1RA and increased secretion of SDF-1α, CXCL13, IL-1RA, IL-15, and IL-21 compared with untreated macrophages. The CCL2/CCR2 axis has been widely implicated in recruitment of monocytes resulting in activation of TAMs and MDSCs, thus regulating immune checkpoint blockade (44–48). In addition, SDF-1α or CXCL12 is also a potent chemoattractant of monocytes resulting in TAMs and contributing to T cell exclusion in PDAC (49). Increased secretion of CCL2 and SDF-1α/CXCL12 by TC-EV macrophages could thus represent an imperative source of these chemokines in the PDAC TME, potentially regulating a positive-feedback loop of monocyte tumor infiltration and subsequent immunosuppressive TAM formation. Our study further highlighted several cytokines and chemokines that can be pro- or antitumoral (IL-1RA, IL-15, IL-21, CXCL13), demonstrating the need to further investigate their role in tumor progression and immune escape, in PDAC, in concert with other secreted factors.

The PDAC immune TME is marked by low levels of effector T cells, an effect largely mediated by immunosuppressive macrophages (50). The M2-like, immunosuppressive phenotype of TC-EV–differentiated macrophages was functionally validated by their impact on T cell proliferation, activation, and antitumor cytotoxicity. Furthermore, these T cells had consistently decreased expression of activation markers CD25 and CD69 as well as costimulation marker CD28, essential for effector T cell activity. Consequently, we demonstrated that T cells in contact with TC-EV–differentiated macrophages are not efficient in killing tumor cells, the same ones that were a source of EVs, highlighting a likely novel mechanism of reduced antitumoral immunity in PDAC via EVs.

In addition to directly influencing infiltrating monocytes, TC-EVs can contact CAFs within the TME and modulate their desmoplastic activity, consequently influencing tumor cells and infiltrating immune cells. Thus, we characterized the ECM produced by CAFs under the influence of TC-EVs and its resulting impact on tumor cell activities as well as monocyte phenotypes and differentiation to macrophages. One of the major components of the ECM is fibronectin, a scaffolding and signaling protein that is highly expressed in the PDAC tumor stroma. Fibronectin is associated with larger tumors and advanced disease and is required for the assembly of other ECM proteins and collagens (51–53). In addition, it promotes tumor cell migration away from the primary tumor site and contributes to the establishment of premetastatic niches in the liver (14, 54). Our present study shows that TC-EVs reduced fibronectin alignment and increased branching in specific patient CAF-derived ECM, potentially indicative of TC-EV–mediated fibronectin remodeling depending on CAF type.

Although fibronectin structural modifications were not consistent between primary CAF samples in our study, all TC-EV–treated CAF–derived ECM resulted in increased migration and invasion of tumor cells, suggesting that factors other than structure were at play in maintaining protumoral aspects of the CAF-derived ECM (55–57). Thus, we evaluated ECM composition following treatment of CAFs with TC-EVs and revealed that TC_1_- and TC_2_-EVs differentially modified CAF matrisome profiles, demonstrating a heterogeneity in the impact of tumor-EVs, potentially due to their modified content. Among pathways and processes highlighted we noticed the presence of members of the WNT pathway, notably SFRP1, not transferred directly from TC-EVs and thus likely produced by CAFs through induction of upstream regulators following EV treatment, then trapped in the ECM. As with other solid cancers, aberrant WNT signaling in PDAC impacts tumor initiation and progression through many aspects such as cell survival, proliferation, differentiation, and drug resistance (58). A subpopulation of CAFs were found to express SFRP1/2 in a SOX2-dependent manner to enhance colorectal cancer cell migration and invasion, in line with our findings (59). To our knowledge, our study is the first to show deposition of ECM with high SFRP1 by CAFs under the influence of TC-EVs, contributing to protumoral actions of the ECM by promoting tumor cell migration and invasion. Functionally, SFRP1 had been long considered as an antagonist of WNT signaling pathway but is now recognized to have functions both as an agonist or an antagonist in a cell type– and concentration-dependent manner. Lower concentrations of SFRP1 in combination with a WNT pathway ligand, WNT3A, were found to stimulate instead of inhibit β-catenin activity, mediated by frizzled-5 receptor. Since SFRP1 is also identified as an actor downstream of activated hedgehog signaling and a modulator of the TGF-β1 pathway (60, 61), further mechanistic studies are required to delineate the specific role of SFRP1 in regulation of these compensatory pathways contributing to tumor cell aggressiveness.

Upon infiltration into tumor tissue, monocytes come into contact with the fibrotic ECM of the PDAC TME. It has been well established that the phenotype and function of various immune cells can be modulated by the characteristics of the ECM. Monocyte-to-macrophage differentiation was found to be impacted by the type of ECM substrate, such as collagen I or fibronectin in physiological and tumor contexts (62, 63), while M2-like polarization of macrophages was described in the presence of hyaluronic acid or internalization of collagen I (64, 65). Since TC-EVs result in protumoral CAF-ECM, we investigated their influence on the differentiation of infiltrating monocytes and resulting macrophages. We observed that ECM produced by CAFs supplemented with recombinant SFRP1 (found enriched in TC-EV–treated CAF–derived ECM) demonstrated the differentiation of healthy monocytes to M2-like, immunosuppressive macrophages. Although Wnt signaling in macrophage differentiation and activity has been extensively studied in various contexts, including cancer, the distinct macrophage polarization in response to canonical versus noncanonical signaling have not yet been established, probably due to the variable repertoire of receptors on monocytes/macrophages and the context of Wnt signaling in presence of additional complementary signaling pathways. Specifically, the role of SFRP1 in immune evasion through its impact on myeloid cells in PDAC is, we believe, unknown, but the emergence of a protumoral SFRP1^+^ CAFs subtypes (59, 66), which we correlated to iCAFs using available public datasets, strengthen its suspected impact on immune evasion. Our study shows a potentially novel role of SFRP1 on myeloid cell differentiation in presence of CAF-derived ECM, resulting in reduced T cell activity. To further expand our findings, knockout of specific Wnt ligands or receptors on monocytes would highlight the mechanism by which Wnt ligands and their antagonists can mediate monocyte-to-macrophage differentiation and polarization.

This proof-of-concept study highlights dual mechanisms of TC-EVs on shaping the immunosuppressive landscape in PDAC, through direct and indirect influence on monocytes. TC-EVs drive monocyte-to-macrophage Alox15b^+^ polarization, leading to T cells’ inactivation (direct effect). In parallel, TC-EVs induce CAFs’ reprogramming, leading to SFRP1-enriched ECM secretion, which consequently drives monocyte-to-macrophage polarization and T cells’ inactivation (indirect effect). Expanding such findings to EVs derived from patient-derived tumor cells or patient-derived organoids could highlight the potentially variable impacts of heterogeneous EV populations on CAF-ECM production and monocyte-to-macrophage differentiation. Considering the critical impact of the immune-suppressive ecosystem in PDAC and its ability to hinder ICI efficacy, unraveling new putative pathways and/or targets influencing such processes is of the greatest importance for patients with PDAC. Hence, our findings suggested that limiting the impact of TC-EVs on monocytes or CAFs is of crucial interest in order to reduce PDAC immunosuppression.

## Methods

### Sex as a biological variable.

Our study examined male and female human PDAC samples. Sex of patients with PDAC was considered and did not appear as a biological variable.

### Cell lines.

Pancreatic epithelial ductal adenocarcinoma cell lines, PANC-1 (TC_1_) and MiaPaCa-2 (TC_2_), were obtained from American Type Culture Collection (ATCC), CRL-1469 and CRL-1420 respectively, and cultured in DMEM Glutamax (Gibco) supplemented with 10% fetal bovine serum (FBS) (Biosera) and 1% antibiotic-antimycotic (Gibco). Nontumor human pancreatic ductal epithelial cell line, hTERT-HPNE or HPDE, was obtained from ATCC, CRL-4023, and maintained in K-SFM supplemented with epidermal growth factor and bovine pituitary extract (Gibco). All cell lines were used for experiments until 20 rounds of passaging. Cell lines were detached using trypsin-EDTA 0.05% (Gibco) following a wash with 1× phosphate-buffered saline (PBS) and split at a 1:10 ratio.

### Primary PDAC CAFs’ isolation and culture.

Small pancreatic tissue blocks were obtained during pancreatic surgery from patients with resectable pancreatic adenocarcinoma at the Institute Paoli-Calmettes. The experimental procedure relating to the use of patient-derived pancreatic tumor pieces was performed after approval from the South Mediterranean Personal Protection Committee, under the reference 2011-A01439-32. Human primary CAFs were isolated as previously described (67). Briefly, the tumors were cut into small pieces of 1 mm^3^ using a razor blade. The tissue pieces were dissociated using the Tumor Dissociation Kit (Miltenyi Biotec) according to the manufacturer’s recommendations. Cells were then resuspended, passed through a cell strainer (100 μM), and finally plated. Human primary CAFs were used between passages 4 and 9. Primary CAF features were verified by flow cytometry with a positive α-SMA and FAP staining. Additional human primary CAFs used were a gift from Elisa Espinet Hernández, German Cancer Research Center (DKFZ, Heidelberg, Germany). All human primary CAFs were cultured in DMEM F-12 medium (Gibco) supplemented with 10% FBS, 1% l-glutamine (Gibco), 1% antibiotic-antimycotic, and 0.5% sodium pyruvate (Gibco). Cells were detached and passaged using StemPro accutase cell dissociation reagent (Gibco).

### Isolation of monocytes and T cells.

Healthy donor blood, in concentrated buffy coats, was obtained from the French Blood Establishment. The blood from a total of 68 healthy donors were used throughout the different experiments in the whole study. PBMCs were isolated using the Ficoll-Paque density gradient method. Briefly, buffy coats were mixed with RPMI 1640 medium (Gibco) supplemented with 10% FBS and 1% antibiotic-antimycotic and centrifuged at 400*g* for 40 minutes (with 0 acceleration and deceleration) with Ficoll-Paque (Cytiva) to obtain a ring of PBMCs. This ring was isolated, incubated for 10 minutes in 1× red blood cell lysis buffer (eBioscience), and washed in 1× PBS 4 times before cell counting. CD14^+^ monocytes were isolated from PBMCs using magnetic labeling and sorting with human CD14 microbeads (Miltenyi Biotec) by positive selection following the manufacturer’s protocol and the autoMacs pro separator (Miltenyi Biotec). Pan T cells were isolated from PBMCs using magnetic labeling and sorting by negative selection with the Pan T cell isolation kit (Miltenyi Biotec) following the manufacturer’s protocol. Purity of isolated monocytes and pan T cells was verified using flow cytometry staining and analysis of CD14 and CD3 marker expression, respectively. Pan T cells were activated and expanded for 48 hours using T cell TransAct, CD3/CD28 stimulation reagent (Miltenyi Biotec), and 40 ng/mL of IL-2 (Miltenyi Biotec).

All cultured cells were tested for mycoplasma contamination using MycoAlert Mycoplasma Detection Kit (Lonza). For treatment with EVs, recipient cells were cultured in EV-free media, where appropriate medium was supplemented with FBS previously ultracentrifuged at 100,000 × *g* for 18 hours to remove FBS-sourced EVs.

### EVs’ isolation and characterization.

EVs were isolated from SN of PANC-1 (TC_1_), MiaPaCa-2 (TC_2_), and HPDE cell lines and primary PDAC organoid cultures as well as their respective cell culture medium as controls. Cancer cell lines, PANC-1 and MiaPaCa-2 cells, were plated once at 70%–80% confluence, and their medium was refreshed 24 hours before collection of SN to DMEM without FBS supplemented with 1% antibiotic-antimycotic. HPDE cell medium was changed to their classic medium, as noted above, at 70%–80% confluence, 24 hours before collection. SN were collected and centrifuged at 500*g* for 5 minutes to remove dead cells and debris. The SN were then centrifuged at 100, 000*g* for 90 minutes at 4°C using the OptimaMax XP Ultracentrifuge (Beckman Coulter). Pellets were collected in 500 μL 1× PBS and loaded onto a qEVoriginal 70 nm column (Izon) to separate vesicles by size-exclusion chromatography. The column was washed with 15 mL of 1× PBS, and 500 μL fractions, fractions 7–10, were collected and pooled. Pooled fractions were washed by centrifugation at 100,000*g* for 90 minutes. Resulting pellets were resuspended in 50–100 μL, depending on the experiment, before treatment. Average cell counts per 10 mL of cell culture SN per 1 T75 flask for EV collection were: 2.19E10^6^ for PANC-1, 5.36E10^6^ for MiaPaCa-2, and 3.89E10^6^ for HPDE. These cell counts allowed for isolation of (average) 1.07E10^9^ EV particles for PANC-1, 5.09E10^8^ for MiaPaCa-2, and 5.84E10^8^ for HPDE. For monocyte EV treatments, 500,000 monocytes were treated with an average of 5.35E10^8^ particles of EVs isolated from 5 mL of cell culture SN from PANC-1, 10 mL from MiaPaCa-2, and 9 mL from HPDE (for the ratio 2:1). For CAF-EV treatments, 100,000 CAFs were treated with an average of 1.07E10^8^ particles of EVs isolated from 1 mL of cell culture SN from PANC-1, 2 mL from MiaPaCa-2, and 1.8 mL from HPDE (for the ratio 2:1). Various ratios 1:1, 2:1, 5:1, and 10:1 were investigated. This ratio was associated with a define number of EVs for PANC-1, and we normalized the experiments with other producing cells by comparing the same number of EVs. This led to a variation in volume used between cell lines but no variation in EVs added during the treatments.

Isolated EVs were characterized for their size and concentration using nanoparticle tracking analysis on the Nanosight NS300 instrument (Malvern Panalytical). Samples were diluted 5-fold in a final volume of 500 μL. Ideal measurement settings and concentrations were found by pretesting the ideal particle-per-frame value (20–60 particles/frame). Camera level was increased and focus adjusted until all particles were visible. For all measurements, three 1-minute videos were captured at 25°C in order to calculate mean values of particle concentration and size. Isolated EVs were further characterized for each cell line using Western blot for detection of classic EV markers: Alix, Tsg-101, syntenin, CD63, CD81, and CD9 (see Table 6 for primary antibody details).

We have submitted all relevant data from our experiments and all information of PANC-1, MiaPaCa-2, and HPDE EVs to the EV-TRACK knowledgebase (EV-TRACK ID: EV260025).

### Treatment of monocytes with EVs and cocultures.

Following CD14^+^ monocyte isolation from PBMCs, monocytes were plated at a density of 500,000 cells per well in 24-well plates in complete RPMI medium containing FBS depleted of EVs. Monocytes were treated with PANC-1, MiaPaCa-2, or HPDE EVs for 2 consecutive days, plated in EV-depleted SN or culture medium (1:2 with RPMI), or treated with 40 ng/mL of M-CSF once (Miltenyi Biotec). Two days following the last EV treatment, differentiated macrophages of all conditions were detached and counted. For recombinant protein treatments, recombinant SFRP1 or Wnt5a was added to wells of monocytes at 1 μg/mL and 500 ng/mL, respectively. For all other assays, macrophages were analyzed by live-cell imaging for viability and phagocytic activity or detached and marked for immunophenotyping by flow cytometry 5 days following the last EV treatment.

For T cell assays, detached macrophages were placed into 1:1 cocultures with same-donor isolated pan-T cells for 3 days. For the T cell proliferation assay, pan-T cells were marked before coculture with CellTrace Violet (Thermo Fisher Scientific). Following coculture, pan-T cells were isolated by recovering cells in suspension, washing wells twice with PBS, and recovering cells from washes. Unstimulated pan-T cells without TransAct and activated T cells with TransAct in simple culture were used as controls. Following 1 wash in complete RPMI medium, pan-T cells were either analyzed by flow cytometry for proliferation assays or counted for subsequent coculture with tumor cells, PANC-1 and MiaPaCa-2, in complete RPMI at a ratio of 1:5 (tumor cell/T cell) for a duration of 3 days.

### Extended methods.

Additional details on reagents, assays, and bioinformatics analysis are described in the Supplemental Methods.

### Statistics.

Statistics were performed on GraphPad Prism 8 for all statistical tests unless otherwise stated below, using 2-tailed unpaired nonparametric Student’s *t* tests with Welch’s correction, mixed effects analysis with uncorrected Fisher’s LSD for multiple comparisons test, or 1-way ANOVA (nonparametric) with Kruskal-Wallis test and Dunn’s multiple comparisons test. For experiments comparing more than 2 conditions, without missing values, we performed 1-way ANOVA test and for experiments comparing more than 2 conditions, with missing values for completely random reason, we performed mixed effects analysis. For those experiments, in figures and legends, asterisks refer to the adjusted *P* value from Dunn’s multiple comparisons test or uncorrected Fisher’s LSD multiple comparisons test while the 1-way ANOVA (Kruskal-Wallis test) and mixed effects analysis *P* value is added directly into the figure. *P* values < 0.05 were considered statistically significant. Data are presented as specified with the figure legends. Statistical analysis of MS data was done with Perseus program (version 1.6.15.0) from the MaxQuant environment. Quantifiable proteins were defined as those detected in above 70% of samples in 1 condition or more. Protein LFQ normalized intensities were base 2–logarithmized to obtain a normal distribution. Missing values were replaced using data imputation by randomly selecting from a normal distribution centered on the lower edge of the intensity values that simulates signals of low abundant proteins using default parameters (a downshift of 1.8 standard deviation and a width of 0.3 of the original distribution). To determine whether a given detected protein was specifically differential, a 2-tailed Student’s *t* test was done using permutation-based FDR-controlled at 0.05 and employing 250 permutations. The *P* value was adjusted using a scaling factor s0 with a value of 1. Analysis was done on biological triplicates, each injected 3 times on mass spectrometers.

### Study approval.

Small pancreatic tissue blocks were obtained during pancreatic surgery from patients with resectable PDAC at the Institute Paoli-Calmettes. The experimental procedure relating to the use of patient-derived pancreatic tumor pieces was performed after approval from the South Mediterranean Personal Protection Committee (Nice, France), under the reference 2011-A01439-32, with written informed consent received.

### Data availability.

All data are available in the main text, supplement, and Supporting Data Values. The MS proteomics data have been deposited to the ProteomeXchange Consortium via the PRIDE partner repository with the dataset identifiers PXD039279 and PXD039439 (68).

## Author contributions

ZH and RT conceived and designed the study. ZH, CM, CR, DB, SST, PF, TB, EL, SA, LC, M Rodrigues, and DI developed the methodology. ZH, CM, CR, DB, SST, PF, FB, SA, M Rodrigues, and DI acquired data (provided animals, acquired and managed cases, and provided facilities). ZH, CM, CR, DB, M Rodrigues, M Rego, LC, PF, FB, GB, and RT analyzed and interpreted data (including statistical analysis, biostatistics, and computational analysis). ZH, M Rego, SV, FB, RT, and SA provided administrative, technical, or material support (including reporting or organizing data and constructing databases). RT supervised the study. ZH, M Rego, and RT wrote the original draft. ZH, CM, M Rego, and RT wrote the revised draft.

## Conflict of interest

The authors have declared that no conflict of interest exists.

## Funding support

- Ministère Français de la recherche, PhD fellowship (to ZH).
- Fondation de France, grant « fundamental and translationnal research on cancer: study of treatment resistance », 00087538 (to TB).
- Fondation de France, grant « fundamental and translationnal research on cancer: study of treatment resistance », 00130850 and 00156332 (to CR).
- The French National Institute of Cancer, INCA, PLBio13-134 and Pancreas 2017 (to SST and RT).
- The fondation ARC pour la Recherche sur le Cancer, ARC PGA 2020 (to SV).
- The fondation ARC pour la Recherche sur le Cancer, ARC Pancreas, ARCPANCREAS2024, 040008193 (to RT).
- The fondation ARC pour la Recherche sur le Cancer, ARC PJA, 20191209372 (to RT).
- The National League Against Cancer, EL-2025.LNCC (to SV).
- The National League Against Cancer, PhD fellowship, TAJG28258 (to DB).
- The National League Against Cancer, PhD Fourth year, TDQA22676 (to ZH).
- The Association Française de Recherche sur le cancer du pancréas, AFRCP (to CM).
- CAPES grant, 88887.466422/2019-00 and 88881.711952/2022-01 (to M Rego).
- CNPq grant, 442179/2023-5, 203087/2019-4, 312426/2021-7 (to M Rego) and 200052/2024-1 (to CM).

## Supplementary Material

Supplemental data

Supporting data values

## Figures and Tables

**Table 6:**
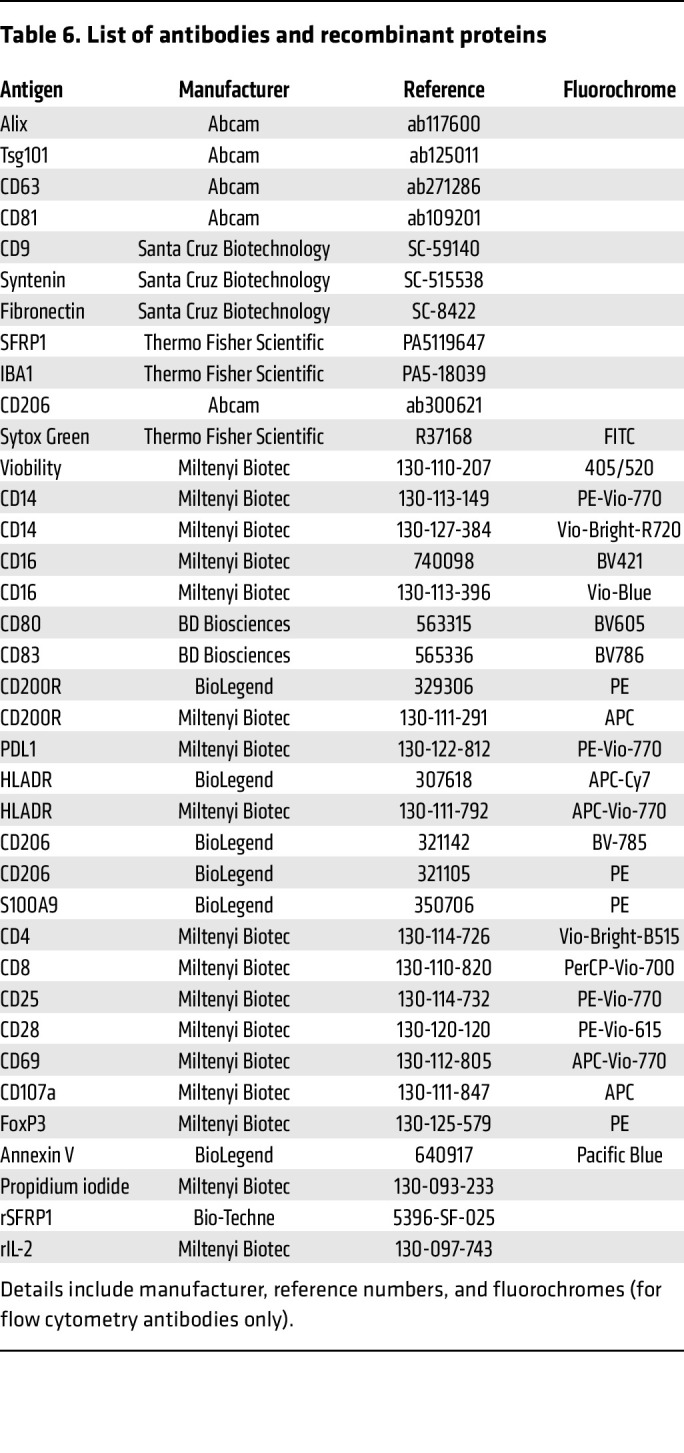
List of antibodies and recombinant proteins

**Table 5:**
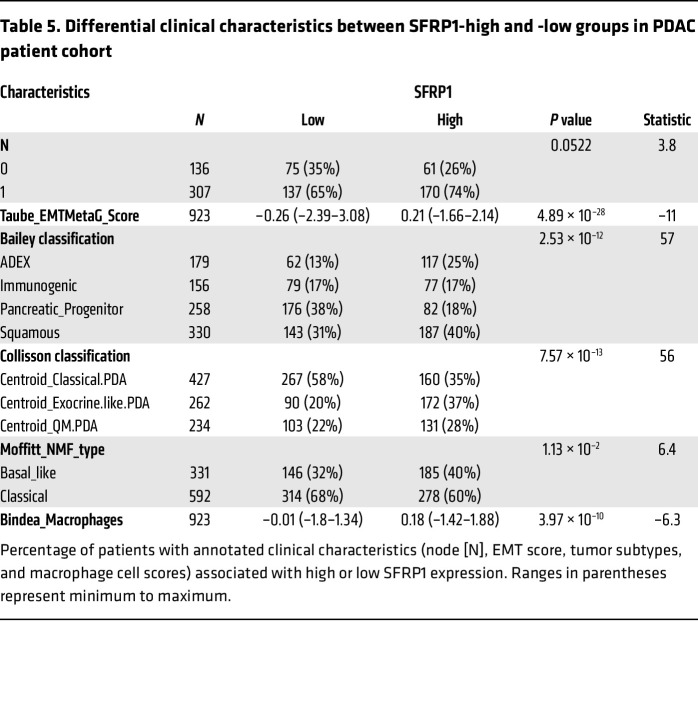
Differential clinical characteristics between SFRP1-high and -low groups in PDAC patient cohort

**Table 3:**
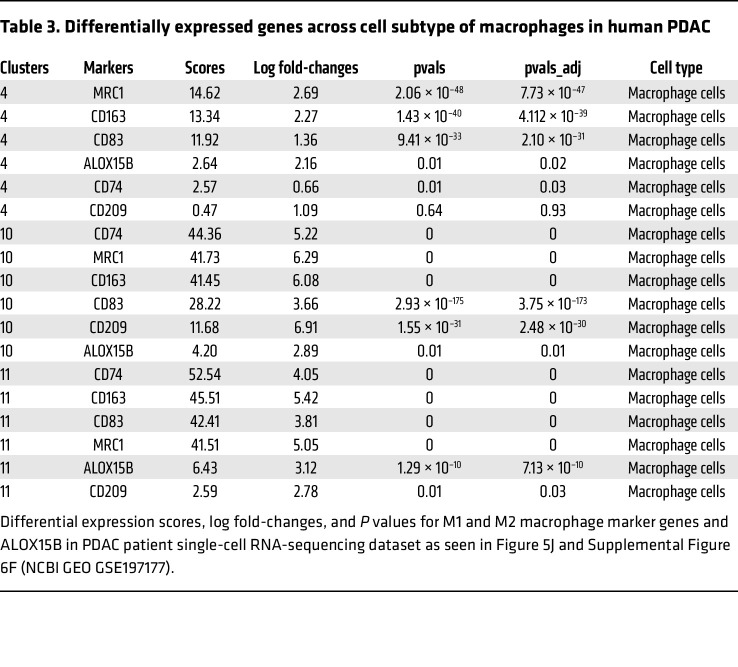
Differentially expressed genes across cell subtype of macrophages in human PDAC

**Table 2:**
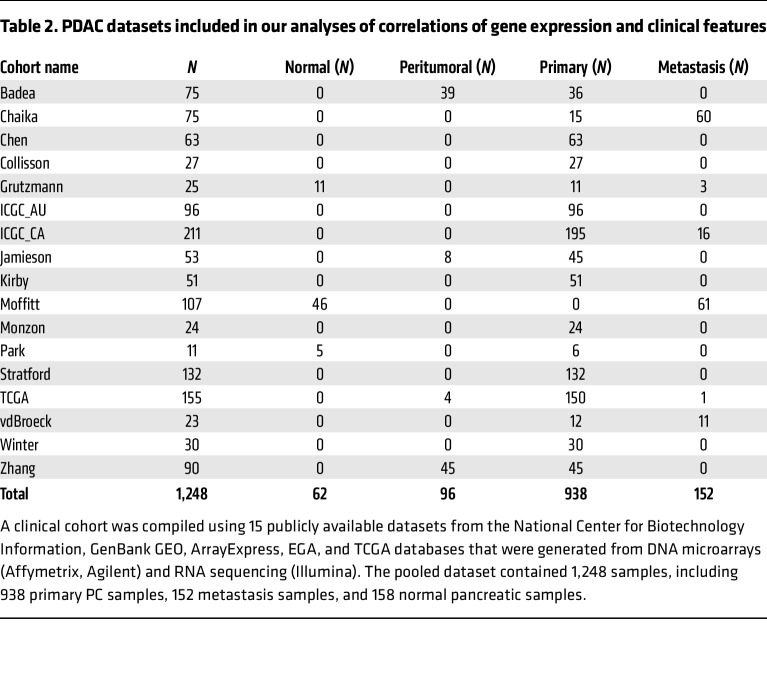
PDAC datasets included in our analyses of correlations of gene expression and clinical features

**Table 1:**
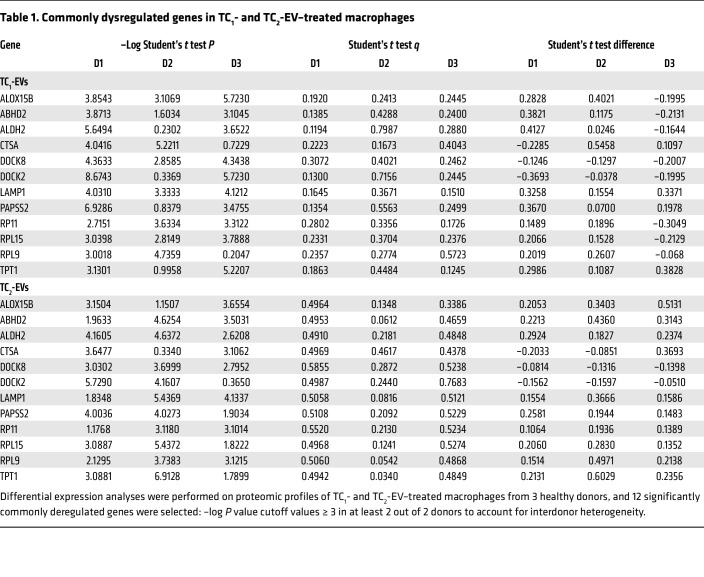
Commonly dysregulated genes in TC_1_- and TC_2_-EV–treated macrophages

**Table 4:**
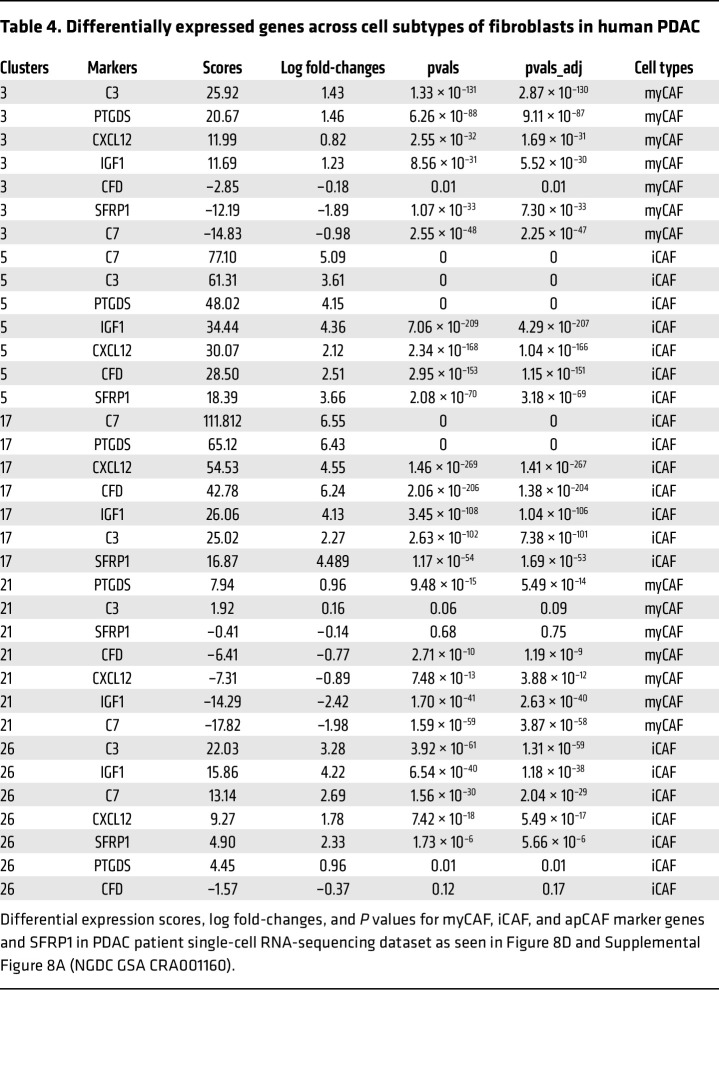
Differentially expressed genes across cell subtypes of fibroblasts in human PDAC

## Version 1 07/02/2026

In-Press Preview

## Version 2 08/17/2026

Electronic publication
