# Supplementary material for: Tumor cell–derived extracellular vesicles foster the immunosuppressive landscape of pancreatic cancer
Source: J Clin Invest. 2026 Jul 2;136(16):e197733. doi: 10.1172/JCI197733 (PMC13476148; doi:10.1172/JCI197733)

Supplemental Figures

Supplemental Figure S1.

(A) Schematic depiction of isolation protocol of EVs from PANC-1 (TC_1), MiaPaCa-2 (TC_2) and HPDE cells. **(B)** Size of EVs (nm) isolated from PANC-1, MiaPaCa-2, and HPDE cells analyzed by nanoparticle tracking analysis (NTA) (n=3). **(C)** Concentration (particles/mL) of EVs isolated from PANC-1, MiaPaCa-2, and HPDE cells analyzed by nanoparticle tracking analysis (NTA) (n=3). **(D)** Representative graphs from NTA analysis of all three cell lines-isolated EVs. **(E)** Representative pictures of electronic microscopy using negative contrast generated by PTA treatment (left panel) and diameter of each single EVs (n=79) measured by Fiji (ImageJ) (middle and right panel). **(F)** Western blot analyses of cell lysates and corresponding isolated EVs expressing EVs markers Alix, Tsg101, CD9, CD63, and CD81. Statistical analyses were performed using unpaired student's t-tests. **(G)** Alive monocytes in percentage (negative for annexin-V or PI) in untreated, HPDE-EVs and escalading doses of 1:1, 2:1, 5:1 and 10:1 TC_1-EVs treated monocytes for 96 hours (N=5).

Supplemental Figure S2.

Multiparametric flow cytometry and phagocytic analysis of TC-EVs differentiated macrophages. **(A)** Violin plots depicting percentage of total cell population expressing monocyte/macrophage markers, M1 macrophage markers, M2 macrophage markers, and MDSC markers in untreated, TC_1-EVs-, TC_2-EVs- and MCSF-treated macrophages. **(B)** MFI of markers described in S2A. **(C)** Representative images of untreated and TC_1-EVs treated macrophages marked with dead-cell marking agent, Cytotox Green (Incucyte) at 30 minutes and 16 hours of incubation. Statistical analyses were performed using Mixed-effects analysis with uncorrected Fisher's LSD for multiple comparisons test (A and B). Significant differences with p-values <0.0001 ****, <0.001 ***, <0.01 **, and <0.05 * are indicated.

Supplemental Figure S3.

(A) Heat maps depicting multiparametric flow cytometry analysis of percentage of total cell populations expressing markers for monocyte/macrophages, M1-macrophages, M2-macrophages, and MDSCs of macrophages differentiated in the following conditions: *TC_1-EVs, TC_2-EVs, HPDE-EVs and corresponding supernatants (SN)-depleted of EVs; DMEM medium-EVs, K-SFM medium-EVs and corresponding SN-depleted of EVs, M-CSF (40ng/mL)* (n=22). **(B)** Heat maps depicting MFI of markers and conditions from S3A (n=22). **(C)** Violin plots of percentage of total cell populations expressing selected markers in conditions described in S3A (n=22). **(D)** Violin plots of MFI of selected markers in conditions described in S3A (n=22).

Supplemental Figure S4.

(A) Representative flow cytometry histograms of Cell-Trace Violet expression in activated T-cells (grey), activated T-cells co-cultured with untreated macrophages (green), and activated T-cells cocultured with TC_1-EVs differentiated macrophages (purple). **(B)** Representative flow cytometry plots of annexin-V and PI expression of TC_1 cocultured with T-cells in conditions described in S4A.

Supplemental Figure S5.

Mass spectrometry analysis of TC_2-EVs differentiated macrophages. **(A)** Venn diagram comparing number of differentially expressed proteins in TC_2-EVs differentiated macrophages with HPDE-EVs differentiated macrophages. **(B)** Heat map of significantly differentially expressed proteins in 3 healthy blood donor-derived monocytes differentiated by TC_2 EVs. **(C)** Volcano plot of significantly up- and down-regulated proteins in TC_2-EVs differentiated macrophages compared to untreated macrophages (n=3). **(D)** Overrepresentation analysis (ORA) tree plot of differentially expressed proteins in TC_2-EVs differentiated macrophages. **(E)** STRING protein interaction network of differentially expressed proteins in TC_2-EVs

differentiated macrophages compared to untreated macrophages. **(F)** Radial plot reporting TC_1-EVs differentiated protein signature and their association with biological pathways. **(G)** Radial plot reporting TC_2-EVs differentiated protein signature and their association with biological pathways.

Supplemental Figure S6.

(A) Box plots of mRNA expression (log2) of 9 out of 12 clinically relevant genes identified in TC-EVs treated macrophage proteomic profile (excluding ALOX15B, RPL11 and DOK8) in PDAC primary samples (N = 938), metastasis samples (N=152) and normal pancreatic samples (N = 158). The orange line represents the median expression level across the whole cohort. **(B)** Table of mRNA expression (log2) and range of 9 out of 12 clinically relevant genes as discretized values in pancreatic samples with indicated p-values (Fischer's exact test). **(C)** Table of ALOX15B, DOCK8, and RPL11 mRNA expression (log2) as discretized values in pancreatic samples with indicated p-values (Fischer's exact test). **(D)** Kaplan-Meier curves of DFS in PDAC patients according to high or low expression of the 9 out of 12 clinically relevant genes identified in TC-EVs treated macrophage proteomic profile. **(E)** Kaplan-Meier curves of OS in PDAC patients in PDAC patients according to high or low expression of the 9 out of 12 clinically relevant genes identified in TC-EVs treated macrophage proteomic profile. **(F)** Correlation of high vs. low expression of ALOX15B with a cell gene signature of macrophages (xCell) in a cohort of PDAC patients (N=469). **(G)** UMAP of leiden clusters and localization and expression of M1 markers (CD74 and CD83) in PDAC patients (GSE197177). **(H)** Statistical evaluation for comparison of prognostic information for OS of ALOX15B expression alone, macrophage score alone, and combination of ALOX15B expression and macrophage score in human PDAC patients using likelihood ratio analysis.

Supplemental Figure S7.

TC-EVs treated CAF-derived ECM Fibronectin fiber lacunarity, length, branchpoints, hyphal growth unit (HGU), and fractal dimension for **(A)** CAF-1 derived ECM and **(B)** CAF-2 derived ECM. **(C)** Mean migration displacement (microns) of TC_1 on ECMs produced by untreated or TC_1-EVs treated CAFs analyzed by time-lapse microscopy (n=3). **(D)** Mean migration speed (microns/second) of TC_1 on ECMs produced by untreated or TC_1-EVs treated CAFs analyzed by time-lapse microscopy (n=3). **(E)** Mean migration distance (microns), of TC_2 on ECMs produced by untreated or TC_2-EVs treated CAFs analyzed by time-lapse microscopy (n=3). **(F)** Invasive capacity, in mean number of invading cells, of TC_2 on ECMs produced by untreated or TC_2-EVs treated CAFs through a Boyden chamber assay (n=3). **(G)** Mean migration displacement (microns) of TC_2 on ECMs produced by untreated or TC_2-EVs treated CAFs analyzed by time-lapse microscopy (n=3). **(H)** Mean migration speed (microns/second) of TC_2 on ECMs produced by untreated or TC_2-EVs treated CAFs analyzed by time-lapse microscopy (n=3). Statistical analyses were performed using unpaired student's t-tests. Significant differences in expression with p-values <0.0001 ****, <0.001 ***, <0.01 **, and <0.05 * are indicated.

Supplemental Figure S8.

(A) Representative confocal microscopy images of fibronectin fibers produced by untreated (Ctr), HPDE-EVs or TC_1-EVs treated primary CAFs derived from patient. (B) Quantification of fibronectin fiber alignment by TWOMBLI (N=3). Statistical analyses were performed using one-way non-parametric ANOVA Kruskal-Wallis test and Dunn's multiple comparisons tests. Significant differences with p-values <0.05 * are indicated. (C) Migratory capacity, in mean migration distance (microns), of TC_1 on ECMs produced by untreated, HPDE-EVs treated or escalading doses of 1:1, 2:1, 5:1 and 10:1 TC_1-EVs treated CAFs analyzed by time-lapse

microscope over 12 hours (N=12). Statistical analyses were performed using one-way non-parametric ANOVA Kruskal-Wallis test and Dunn's multiple comparisons tests. Significant differences with p-values <0.01 **, and <0.05 * are indicated.

Supplemental Figure S9.

Correlation analyses of iCAF, T cells and Macrophages from PDAC patients (CRA001160). **(A)** UMAP of leiden clusters from fibroblast cell type and localization and expression of myCAF markers (MMP11 and POSTN). **(B)** Immunofluorescence images of SFRP1 and a-SMA expression in human PDAC tissue-microarrays. Images taken at 20x (left) and 40x (right) magnification. **(C)** Heatmap of the expression of IGF1 in iCAF and CD69 in T cells from each patient. **(D)** Relative percentage of iCAFs and T cells in each patient. **(E)** Correlation analysis between the percentage of T cells and iCAF in PDAC patients. **(F)** Heatmap of the expression of IGF1 in iCAF and CD163 in macrophage from each patient. **(G)** Correlation analysis between the percentage of CD163 in macrophage and IGF1 in iCAF.

Supplemental Methods

Primary PDAC CAF-derived ECM production

To produce CAF-derived ECMs, 12-well plate wells or 24-well Boyden chamber inserts (for subsequent cell culture) and cell culture chamber slides (for immunofluorescence) were pre-treated by coating with 0.2% gelatin for 1 hour, crosslinking of gelatin by 1% glutaraldehyde for 30 minutes, and quenching of glutaraldehyde by 1M glycine for 30 minutes. Before cell plating, the wells were incubated with primary CAF medium for 30 minutes. Primary CAFs were plated at 100% confluency, approximately 200, 000 cells in a 12-well plate. Following 24 hours of plating, primary CAF medium was changed to CAF-EVs depleted medium supplemented with 50μg/mL of L-ascorbic acid (Sigma-Aldrich). Medium was refreshed every 48 hours for a total duration of 9 days, starting from the first medium change, to allow for ECM production. CAFs were treated with tumor-cell derived EVs, during 2 consecutive days, for a total of 4 treatments, twice during the 9-day period. Decellularization of CAF-derived ECM was performed by incubation with extraction buffer (20mM ammonium-hydroxide, 0.5% Triton-X100) for 5 minutes at 37°C and 5% CO_2 until no living cells could be observed. The decellularized ECMs were washed three times with 1x PBS carefully to not dislodge the matrix from the well. For further incubation of tumor cells or monocytes on CAF-derived ECMs, DNA degradation was performed by incubating 15μg/mL of DNase I (Roche) in PBS supplemented with Ca^{2+}/Mg^{2+} for 30 minutes at 37°C and in 5% CO_2. ECMs were washed twice with PBS supplemented with Ca^{2+}/Mg^{2+} and incubated in appropriate cell culture medium, either for tumor cells or monocytes, for 30 minutes before subsequent cell plating. For recombinant protein treatments, recombinant SFRP1 or Wnt5a was added to wells of monocytes at 1μg/mL and 500 ng/mL respectively.

Migration and invasion assays on ECM

Migratory capacity of tumor cells (TCs), PANC-1 and MiaPaCa-2, on CAF-derived ECMs was measured using live-cell tracking in time-lapse microscopy. Tumor cells were either labelled with Incucyte™ rapid nuclight rapid red dye (MiaPaCa-2) at a 1:500 dilution following the manufacturer's protocol (Sartorius) or were transfected with DsRed lentivirus (PANC-1). 10, 000 tumor cells were plated on ECMs in a 12-well plate in their classic medium and incubated overnight before analysis. The following day, phase and fluorescence images were taken every 15 minutes at randomly-chosen locations in the well using a time-lapse microscope (Zeiss-Roper Scientifics) or the Incucyte® S3 live-cell analysis system (Sartorius) for 12 hours. Analysis was performed using the TrackMate plugin on ImageJ Fiji using the following

parameters: detection by LoG detector with an estimated diameter of 25 microns and LAP tracker with gap-closing detection. Dying cells, dividing cells, or cells that were not in the field-of-view for the entire 12-hour tracking period were eliminated from the analysis. Resulting data on migration distance, displacement, speed and directionality were collected.

Invasive capacity of TCs, PANC-1 and MiaPaCa-2, was measured using the Boyden chamber assay. CAF-derived ECMs were produced in 8-micron 24-well PET membrane inserts (Corning), decellularized and treated as previously described. 10,000 TCs were plated on CAF-derived ECMs and incubated for 24 hours. The inserts were then washed with 1x PBS once and remaining cells in the upper-part of the insert were removed using a cotton-swab. Inserts were fixed with 4% paraformaldehyde for 15 minutes at room temperature and washed three times with 1x PBS. Inserts were incubated in 0.5% crystal violet staining solution for 20 minutes at room temperature. Inserts were then washed three times with 1x PBS and left to dry overnight. The following day, 10 photos per insert were taken at 10x magnification using the EVOS FL cell imaging system (ThermoFisher Scientific). Invaded cells were counted using cell counter on ImageJ Fiji.

Sartorius/Incucyte Live Imaging

Viability assay

To measure viability of macrophages differentiated by TC-EVs, live cell imaging was performed with the Incucyte S3 live-cell analysis system (Sartorius). Following 5-day incubation with TC-EVs, macrophages at 30% confluency were incubated with 250nM of Incucyte® cytotox green dye (Sartorius). The green dye enters cells upon compromise of plasma membrane integrity and binding to DNA. Images were taken with the following parameters: two images per well (3 wells per condition), 10x magnification, 350ms acquisition time, every 2 hours for a duration of 3 days. Integrated adherent cell-by-cell analysis was performed to calculate total green integrated intensity, representative of dying or dead cells, which was normalized to cell confluency and used to calculate viability of macrophages.

Phagocytosis assay

To measure phagocytic capacity of macrophages differentiated by TC-EVs, live cell imaging was performed with the same system as described above. Following 5-day incubation with TC-EVs, macrophages at 30% confluency were incubated with 10μg of IncuCyte® pHrodo® green E. coli Bioparticles®. Phagocytosis is measured by detection of fluorescence in cells when E. coli bioparticles are engulfed by macrophages and enter their acidic phagosomes, leading to an increase in fluorescence. Images were taken with the following parameters: two images per well (3 wells per condition), 10x magnification, 400ms acquisition time, every 15 minutes for a duration of 3 hours. Integrated adherent cell-by-cell analysis was performed to calculate total green integrated intensity, representative of phagocytosing cells, which was normalized to cell confluency and used to calculate phagocytic capacity of macrophages.

Tumor cell killing/cytotoxicity assay

To measure the cytotoxicity of pan T-cells cocultured with TC-EVs differentiated or untreated macrophages against tumor cells, live-cell imaging was performed with the same system as described above. 5000 tumor cells, PANC-1 or MiaPaCa-2 were seeded in 96-well plates in their classic medium and left to adhere overnight. The following day, pan T-cells were removed from the 72-hour coculture with macrophages, counted, and plated at a ratio of 5:1 with tumor cells in RPMI complete medium with a final concentration of 5μM of Incucyte® Caspase-3/7 green dye. Images were taken with the following parameters: three images per well (3 wells per condition), 10x magnification, 350ms acquisition time, every 2 hours for a duration of 3 days. Integrated adherent cell-by-cell analysis was performed to calculate total green integrated intensity, representative of cells in apoptosis, which was normalized to cell confluency and used to calculate apoptotic activity of tumor cells. Adherent cell-by-cell analysis settings used are as

follows; under cell boundary: segmentation adjustment at 0.7, hole fill adjusted size in pixels at -8, under seed: cell detection sensitivity set at 1.1, cell contrast at 3 (high), and cell morphology at 3, under cell-by-cell: minimum area set to 300µm^2. These parameters allowed the elimination of T-cells from detection of green fluorescence and apoptotic activity.

Confocal Microscopy

CAF-derived ECM were imaged using a confocal Zeiss LSM 880 microscope (Carl Zeiss Microscopy) in z-stacks and optimal pixel densities, equipped with a 63x NA 1.4 objective and a GaAsP detector using Zen software. Images were analyzed using FIJI software, and a maximum intensity projection over the Z axis was performed.The Worklfow Of Matrix BioLogy Informatics (TWOMBLI) plugin was used for the quantification of ECM patterns[1].

Electronic Microscopy

5µL of EV samples are deposited for 8 minutes on a glow discharged Formvar grid of 200 mesh (74µm). After washed with distilled water (DW), the grid is treated for 30 seconds with 2% phosphotungstic acid (PTA), and dried. EVs are then visualized using a Tecnai G2 (Thermofischer, USA) at 200 KeV with a camera Olympus (Japan).

Western Blot

Cells or CAF-derived ECMs were lysed using a Laemmli lysis buffer 3X (6% SDS, 30% Glycerol and 187.5mM Tris-HCl, pH 6.8). Samples were sonicated for 30 seconds with alternating 1 second pulse on/off at a frequency of 20 kHz before boiling them at 95°C for 5 minutes. Protein dosing was performed using the BCA assay and 10% SDS-PAGE gels were loaded with equal concentrations of proteins for each condition, 30 or 40µg of protein per well depending on the experiment. Appropriate concentrations of protein samples were mixed with 4x reducing lamelli buffer (250mM Tris-HCl, 8% SDS, 40% Glycerol, 5% β-merceptoethanol, 0.02% Bromophenol blue) and boiled at 95°C for 5 minutes before loading, except for EVs where the entire EVs pellet was directly resuspended in 1x lamelli buffer before loading. Electrophoretic separation of proteins was performed by application of 110V for 75 minutes. Separated proteins were then transferred to a 0.2µm nitrocellulose membrane using the TransBlot turbo transfer system (Bio-Rad) and protein transfer was verified by Ponceau S staining. Membranes were blocked for non-specificity using either 5% non-fat dry milk powder in TBS (Tris Buffered Solution)-Tween 0.05% or blocking buffer (BSA 30g/L, Porcine Gelatin 2.5g/L, Tween20 0.1%, 1mM EDTA, 150mM NaCl, 10mM Tris, in H2O, pH 7.4) for 1 hour before incubation with primary antibodies in blocking solution overnight at 4°C (Table 1). Proteins were detected using HRP-conjugated secondary antibodies. Protein bands were quantified using ImageJ Fiji software and normalized to housekeeping protein vinculin for cell lysates.

Flow cytometry

Cells were detached and washed once with 1x PBS and resuspended in FACs buffer (0.5% BSA, 2mM EDTA in PBS). For immunophenotyping of macrophages and T-cells, the methods used are as follows. Viability staining was performed using either a fixable viability dye, Viobility dye 405/520, according to manufacturer's instructions (Miltenyi Biotec) or by adding one drop of Sytox green flow reagent (ThermoFisher Scientific) to samples prior to analysis. Compensation was performed using either Ultracomp eBeads plus compensation beads (Invitrogen) or the anti-REA MACs comp bead kit (Miltenyi Biotec). Staining with extracellular fluorochrome coupled-antibodies was carried out in 100uL of FACs buffer for 20 minutes in the dark at 4°C. For immunophenotyping of TC-EVs differentiated macrophages or T-cells, macrophages were incubated with extracellular antibodies (Table 1).

For fluorescent labeling of EVs and subsequent analysis of cellular uptake of EVs by CAFs and monocytes, EVs were isolated as previously described and labeled with PKH67 green fluorescent cell linker kit (Sigma-Aldrich) according to the manufacturer's protocol. For

Annexin-V/propidium iodide (PI) staining, cells were resuspended in 1x binding buffer (10 mM Hepes adjusted to pH 7.4, 140 mM NaCl and 2.5 mM $CaCl_2$) and stained with Annexin-V (Biolegend) and PI (Miltenyi Biotec) for 20 minutes at room temperature in the dark. For proliferation analysis, CellTrace violet dye (ThermoFisher Scientific) was resuspended in DMSO, added to pan T-cells in PBS at a 1:1000 dilution and incubated for 20 minutes at 37°C. Following incubation, five times the original staining volume of complete RPMI medium was added and left for 10 minutes before two washes with complete RPMI medium to remove the excess dye. All samples were rewashed in FACs buffer once before being analyzed using a BD LSR-Fortessa X20 cell analyzer. Data were analyzed using the FlowJo software (BD Biosciences).

Clinical correlation analyses

We gathered clinicopathological and gene expression data of clinical pancreatic samples from 15 publicly available datasets (Table 2). Data were collected from the National Center for Biotechnology Information (NCBI)/Genbank GEO, ArrayExpress, EGA, and TCGA databases and had been generated using DNA microarrays (Affymetrix, Agilent) and RNA-seq (Illumina). The pooled dataset contained 1248 samples, including 938 primary PC samples, 152 metastasis samples, and 158 normal pancreatic samples. A total of 754 primary PC samples were informative for DFS and 755 for OS. Data analysis required preanalytic processing as previously published[2]. Expression of selected genes in primary tumors was measured as a discrete value by using the median expression level as cut-off and defined two tumor classes thereafter designated "high" and "low". The molecular subtype of tumors (classical *versus* basal-like) was determined by applying to each sample in each dataset separately the Moffitt[3] multigene classifier. Correlations between ALOX15B-high *versus* ALOX15B-low tumor classes and clinicopathological features were analyzed using the t-test or the Fisher's exact test when appropriate. DFS was calculated from the date of diagnosis to the date of relapse or death from PC. OS was calculated from the date of diagnosis to the date or death from PC. Follow-up was measured from the date of diagnosis to the date of last news for event-free patients. Survivals, calculated using the Kaplan-Meier method, were compared with the log-rank test. Univariate and multivariate DFS analyses were done using Cox regression analysis (Wald test). Variables tested in univariate analyses included patients' age at diagnosis (>60 *versus* ≤60 years), sex, pathological tumor size (pT3-T4 *versus* pT1-T2), pathological lymph node status (positive *versus* negative), anatomic location (head *versus* body/tail), pathological type (non-ductal *versus* ductal), Moffitt's molecular subtypes (classical *versus* basal-like), and "high" *versus* "low" gene expression. Variables with a *P*-value <0.05 were tested in multivariate analysis. The likelihood ratio (LR) tests were used to assess the prognostic information provided by ALOX15B expression alone, the Bindea's macrophage signature alone and both of them combined, assuming a X2 distribution. Changes in the LR values (LR-ΔX2) quantified the relative amount of information of one model compared with another one. All statistical tests were two-sided at the 5% level of significance. Statistical analysis was done using the survival package (version 3.1-12) in the R software (version 3.5.2; www.cran.r-project.org/).

Single cell RNA sequencing analyses

Datasets

To investigate CAFs populations, we utilized scRNA-seq data from 24 PDAC patients available in the NGDC database (CRA001160) (https://ngdc.cncb.ac.cn/gsa/browse/CRA001160). For macrophage populations, we used scRNA-seq data from three PDAC patients data from Zhang et al (GEO accession: GSE197177)[4].

Data preprocessing

All single cell analyses were conducted using the SCANPY package[5], within Jupyter Notebook (Python 3.9.18). We applied cell and gene filtering with the following criteria: cells with ≥ 200 detected genes, genes expressed in at least 3 cells. Cells were also filtered based on

mitochondrial gene content, removing those with >6% (for CAFs) or >20% (for macrophages) mitochondrial transcripts. Following the filtering, Following the filtering, the data were normalized to the total expression, multiplied by a scaling factor of 10,000, and log-transformed.

Cell Clustering and Dimensionality Reduction

To identify cell clusters, we followed the default SCANPY workflow. Firstly, we identify highly-variable genes. We regressed the unwanted sources of variation (total counts and mitochondrial percentage). The data were scaled to unit variance and zero mean. We perform Principal Component Analysis (PCA), retaining the first 25 (for CAF dataset) or 30 (for macrophage dataset) principal components (PCs). We computed the nearest neighbor graph and constructed a UMAP embedding for visualization. Cells were clustered using the Leiden algorithm, and differentially expressed genes were identified using the T-test method.

Dataset Integration

For integration of macrophage datasets, we first filtered for common genes across the three samples. Then we used the scanpy.tl.ingest function to align the embeddings and annotations based on the cluster information. After integration, the objects were merged into a new dataset.

Cell annotation

CAFs

For the annotation of CAF populations, we utilized the cell type annotation for the CRA001160 dataset[6]. We then clusterized the fibroblast population and analyzed their localization within clusters using established iCAF (C3, C7, CFD, PTGDS, CXCL12 and IGF1) and myCAF (MMP11 and POSTN). Differentially expressed genes (DEGs) within these clusters further validated the classification of CAF subtypes.

Macrophages

To identify macrophage populations in the GSE197177 dataset, we applied the decoupleR package[7] to perform Over-Representation Analysis (ORA). The top three most likely cell type annotations for each cluster were determined based on ORA scores. Final annotations were refined using the static values of mean change and p-adjusted. To further confirm macrophage annotation, we used the M2 markers (MRC1, CD209 and CD163), applying the same validation approach used for CAF populations.

Mass spectrometry

Matrisome

Proteomic analysis of decellularized ECMs was performed as described: Briefly, ECM proteins in culture wells were solubilized in 8M urea, reduced with 10mM DTT (56°C, 45mn) and alkylated with 50 mM iodoacetamide (30mn, RT). Proteins were thereafter digested with first PNGase F (1500 units/ml, 37°C, 2h, New England BioLabs), endoproteinase Lys-C (1µg/ml, 37°C, 2h, Promega) and high-sequencing-grade trypsin (2µg/ml, 37°C, 16h, Promega). Peptides were desalted on Sep-Pak C18 ligh-cartridge (Waters), dried under speed vacuum and were reconstituted with 15µl 0.1% trifluoroacetic acid in 2% acetonitrile for LC-MSMS analysis. Each biological condition was prepared in triplicates and further injected thrice for

LC-MS/MS analysis using an Orbitrap Fusion Lumos Tribrid Mass Spectrometer (ThermoFisher Scientific, San Jose, CA) online with a nanoRSLC Ultimate 3000 chromatography system ThermoFisher Scientific, Sunnyvale, CA). 1µl corresponding to 7 % of the sample was injected. First, peptides were concentrated and purified on a pre-column PepMap100 C18, 2cm × 100µm I.D, 100Å pore size, 5µm particle size in solvent A (0.1% formic acid in 2% acetonitrile). In the second step, peptides were separated on a reverse phase LC EASY-Spray C18 column PepMap RSLC C18, 50cm × 75µm I.D, 100Å pore size, 2µm particle size (ThermoFisher) at 300nL/min flow rate and 40°C. After column equilibration using 4% of solvent B (20% water - 80% acetonitrile - 0.1% formic acid), peptides were eluted from the analytical column by a two-step linear gradient (2-20% acetonitrile/H2O; 0.1% formic acid for 90min and 20-45% acetonitrile/H2O; 0.1% formic acid for 20min). For peptide ionization in the EASY-Spray nanosource in front of mass spectrometer, spray voltage was set at 2.2 kV and the capillary temperature at 275 °C. The Orbitrap Lumos was used in data dependent mode to switch consistently between MS and MS/MS. Time between Masters Scans was set to 3 seconds. MS spectra were acquired with the Orbitrap in the range of m/z 400-1600 at a FWHM resolution of 120 000 measured at 400 m/z. AGC target was set at 4.0e5 with a 50 ms Maximum Injection Time. For internal mass calibration the 445.120025 ions was used as lock mass. The more abundant precursor ions were selected and collision-induced dissociation fragmentation was performed in the ion trap to have maximum sensitivity and yield a maximum amount of MS/MS data. Number of precursor ions was automatically defined along run in 3s windows using the "Inject Ions for All Available parallelizable time option" with a maximum injection time of 300 ms. The signal threshold for an MS/MS event was set to 5000 counts. Charge state screening was enabled to exclude precursors with 0 and 1 charge states. Dynamic exclusion was enabled with a repeat count of 1 and duration of 60 s.

EVs-differentiated macrophage proteomics

Blood from 3 donors (D1, D2, D3) were used for this experiment, each treated in triplicate with PANC-1 (PE), MiaPaCa-2 (M), healthy pancreatic ductal epithelial (HPDE)(H) EVs or PBS (C: Control vehicle). For relative proteomic analysis, proteins from cell pellets were solubilized in 15µl of LDS sample buffer (2x concentrated), (Invitrogen, Life Technologies) before loading on NuPAGE™ 4–12% Bis–tris acrylamide gels according to the manufacturer's instructions (Invitrogen, Life Technologies). Running of samples was stopped as soon as proteins stacked as a single band and following imperial blue staining (Life Technologies), the upper part of the gel containing the proteins was cut and processed for classical in gel digestion (washes, thiols reduction with 10 mM DTT and cystein alkylation with 55 mM iodoacetamide. Each band was further digested as previously described with trypsin and analyzed by liquid chromatography (LC)-tandem MS (MS/MS) using an Orbitrap Fusion Lumos Tribrid Mass Spectrometer (ThermoFisher Scientific, San Jose, CA) online with a nanoRSLC Ultimate 3000 chromatography system (ThermoFisher Scientific, Sunnyvale, CA). First, peptides were concentrated and purified on a pre-column PepMap100 C18, 2cm × 100µm I.D, 100Å pore size, 5µm particle size in solvent A (0.1% formic acid in 2% acetonitrile). In the second step, peptides were separated on a reverse phase LC EASY-Spray C18 column PepMap RSLC C18, 50cm × 75µm I.D, 100Å pore size, 2µm particle size (ThermoFisher) at 300nL/min flow rate and 40°C. After column equilibration using 4% of solvent B (20% water - 80% acetonitrile - 0.1% formic acid), peptides were eluted from the analytical column by a two-step linear gradient (2-20% acetonitrile/H2O; 0.1% formic acid for 90min and 20-45% acetonitrile/H2O; 0.1% formic acid for 20min). For peptide ionization in the EASY-Spray nanosource in front of mass spectrometer, spray voltage was set at 2.2 kV and the capillary temperature at 275 °C. The Orbitrap Lumos was used in data-independent mode with the following parameters. First, MS spectra were acquired in the Orbitrap in the range of m/z 375-1500 at a FWHM resolution of 120,000 measured at 400 m/z. AGC target was set at standard parameters with an automatic Maximum Injection Time. MS2 spectra were acquired in the Orbitrap with a resolution of 30,000, in the mass range of 200-1800 m/z after isolation of parent ion in the quadrupole and

fragmentation in the HCD cell under collision Energy of 30%. DIA parent ion range was from 400 to 1000 m/z divided into 40 windows 16 Da wide and from 1000 to 1500 m/z divided into 10 windows 50 Da wide.

Data Processing Protocol of Matrisomes

Relative intensity-based label-free quantification (LFQ) was processed using the MaxLFQ algorithm from the freely available MaxQuant computational proteomics platform, version 1.6.3.4. Spectra were searched against the Human database extracted from UniProt on the 24th of April 2021 and containing 20395 entries (reviewed). The following parameters were used for searches: (i) trypsin allowing cleavage before proline; (ii) two missed cleavages were allowed; (ii) monoisotopic precursor tolerance of 20 ppm in the first search used for recalibration, followed by 4.5 ppm for the main search and 0.5 Da for fragment ions from MS/MS ; (iii) cysteine carbamidomethylation (+57.02146) as a fixed modification and methionine, proline oxidation (+15.99491), asparagine deamination (+0.984016) and N-terminal acetylation (+42.0106) as variable modifications; (iv) a maximum of five modifications per peptide allowed; and (v) minimum peptide length was 7 amino acids and a maximum mass of 4,600 Da. The match between runs option was enabled to transfer identifications across different LC-MS/MS replicates based on their masses and retention time within a match time window of 0.7 min and using an alignment time window of 20 min. The quantification was performed using a minimum ratio count of 1 (unique+razor) and the second peptide option to allow identification of two co-fragmented co-eluting peptides with similar masses. The false discovery rate (FDR) at the peptide and protein levels were set to 1% and determined by searching a reverse database. For protein grouping, all proteins that cannot be distinguished based on their identified peptides were assembled into a single entry according to the MaxQuant rules.

Data Processing Protocol of EVs-differentiated macrophages proteomics

Relative intensity-based label-free quantification (LFQ) was processed using the DIA-NN 1.8 algorithm. Raw files were searched against the Human database extracted from UniProt on the 2th of February 2022 and containing 20395 entries (reviewed) with the addition of a protein contaminant bank[8]. The following parameters were used for searches: (i) trypsin allowing cleavage before proline; (ii) one missed cleavages were allowed; (iii) cysteine carbamidomethylation (+57.02146) as a fixed modification and methionine oxidation (+15.99491) and N-terminal acetylation (+42.0106) as variable modifications; (iv) a maximum of 1 variable modification per peptide allowed; and (v) minimum peptide length was 7 amino acids and a maximum of 30 amino acids. The match between runs option was enabled to transfer identifications across different LC-MS/MS replicates based on their masses and retention time. The precursor false discovery was set to 1%. DIA-NN parameters were set on Single-pass mode for Neural Network classifier, Robust LC High precision for quantification strategy and RT-dependent mode for Cross-run normalization. Library was generated using Smart profiling set up. Main output file from DIA-NN was further filtered at 1% FDR and LFQ intensity was calculated using the iq package at 1% q-value (Bioinformatics 2020;36(8):2611-2613. iq: an R package to estimate relative protein abundances from ion quantification in DIA-MS-based proteomics; Thang V Pham, Alex A Henneman, Connie R Jimenez).

Data analyses of proteomics

Extraction of validated genes by Mass Spec on the three donors and selection of validated genes in at least two of the donors. Genes with a difference in abundance (-log10(p-value)>3 or p-value<0.001) were considered as potential targets. Targets also validated in HPDE were

excluded. 76 targets were selected for TC_1 (PANC-1) and 54 for TC_2 (Miapaca-2. Protein networks associated with the identified gene intersections were generated by STRING (v12) on https://string-db.org/. Volcano plots and heatmaps were carried out in Rstudio 4.4[9] using the EnhancedVolcano[10] (v1.22.0), ComplexHeatmap (v.2.20)[11,12] and ggplot2 (v.3.5.1) packages. For TC_1 (PANC-1), we performed Gene Set Enrichment Analysis of Gene Ontology (gseGO) using clusterProfiler (v4.12.6)[13] package.

Pathway and Process Enrichment Analysis of the targets was performed on metascape v3.5.2023101 on https://metascape.org/. 41 targets for TC_2 (Miapaca-2) were retained after enrichment was distributed in the Top 10 clusters. The radial table of the cluster genes intersections was generated using Circos Table Viewer software (v0.63-10)[14] on http://mkweb.bcgsc.ca/tableviewer/ .

Availability of EVs-related Protocols

Written details on experimental procedures have been submitted to the Transparent reporting and Centralizing Knowledge in Extracellular Vesicle Research (EV-TRACK) knowledgebase (EVTRACK ID : EV260025) that allows visualization and interpretation of EV Metrics then access to the according study report.

Cytokine quantification by array

Conditioned medium of untreated and TC_1-EVs -treated primary CAFs, seeded at $1x10^6$ cells in 100mm petri dishes, were collected 48h following treatment, centrifuged at 500xg for 5 minutes to remove cells and debris, aliquoted and frozen at -80°C until use. Samples were thawed on ice and cytokine quantification was performed using the human cytokine antibody array kit (Abcam, ab133998) following the manufacturer's protocol. Cytokines were detected by chemiluminescence and quantified using the protein array analyzer for ImageJ. Standard dots were used to normalize and remove background.

Multiplex ELISA

For multiplex ELISA analysis of conditioned medium of monocyte-derived macrophages, conditioned medium of untreated and EV-treated macrophages was collected and centrifuged at 250xg for 5 minutes to remove cells and debris. Conditioned media were aliquoted and stored at -80°C until use. They were thawed on ice and analyte expression was assessed using a custom Human Procartaplex mix-and-match 32-plex panel following the manufacturer's protocol (ThermoFisher). The panel included: BLC (CXCL13), CD40L, Eotaxin-2 (CCL24), Eotaxin-3 (CCL26), Fractalkine (CX3CL1), GROα (CXCL1), IFN gamma, IL-1 beta, IL-1RA, IL-6, IL-8 (CXCL8), IL-10, IL-13, IL-15, IL-21, IL-23, IP-10 (CXCL10), MCP-1 (CCL2), MCP-3 (CCL7), MCP-4 (CCL13), MDC, MIG (CXCL9), MIP-1α (CCL3), MIP-3α (CCL20), MMP-1, RANKL, RANTES (CCL5), SDF-1α , TARC (CCL17), TNFα, VEGF-A, VEGF-D. Optical density measurement of plates was performed using the Bioplex 200 (BioRad). Analytes with measurements exceeding or below the values of the standard curve were excluded from analysis.

Immunohistochemistry

A total of 4µm formalin-fixed, paraffin-embedded human Tumor Micro Array (TMA) pancreas sections were deparaffinized in xylene and rehydrated through a graded ethanol series. An antigen retrieval step (10 mM sodium citrate, 0.05% Tween 20, or TRS9 (S236784-2, Agilent Technologies) or TRS6 (S169984-2) at 95°C) was performed before quenching endogenous peroxidase activity [3% (v/v) H2O2]. Tissue sections were then incubated with antibodies, and

immunoreactivity was visualized using the Polink 2 Plus HRP Mouse with DAB Kit (GBI Labs, D37-6) or Polink 2 Plus HRP Rabbit with DAB Kit (GBI Labs, D39-6) according to the manufacturers' protocols. Counter staining with Mayer hematoxylin was followed by a bluing step in 0.1% sodium bicarbonate buffer before final dehydration, clearance, and mounting of the sections. Primary antibodies used in the above experiments are listed in Table 1.

Immunofluorescence

For immunofluorescence of CAF-derived ECM, following decellularization and washes, ECMs were incubated in 4% paraformaldehyde for 15 minutes at room temperature. ECMs were washed three times with 1x PBS and blocked for non-specific binding using 1% bovine-serum albumin (BSA) in PBS for 1 hour at room temperature. Primary antibodies, anti-fibronectin at a1:200 dilution (Santa Cruz Biotechnology), anti-SFRP1 at a 1:500 dilution (Invitrogen), anti-Wnt5a at a 1:200 dilution (Invitrogen), were incubated in 1% BSA/PBS overnight in a humid chamber at 4°C. The following day, ECMs were washed three times with 1x PBS and incubated with appropriate species-specific fluorochrome-coupled secondary antibodies (Alexa-Fluor 488, Alexa-Fluor 568) in 1% BSA/PBS in a humid chamber for 2 hours. Slides were mounted with Ibidi mounting medium (Ibidi) for non-detachable chambers and ProLong gold antifade mountant (ThermoFisher) for detachable chambers before analysis by confocal microscopy.
For immunofluorescence of tissues, the methods used are as follows. A total of 4µm formalin-fixed, paraffin-embedded human Tumor MicroArray (TMA) pancreas sections were deparaffinized in xylene and rehydrated through a graded ethanol series. An antigen retrieval step (10 mM sodium citrate, 0.05% Tween 20, or TRS9 or TRS6 at 95°C (Agilent Technologies)) was performed before tissue sections were preincubated in blocking solution (PBS, 0.1% Triton X-100, 10%[v/v] donkey serum) for 1 hour at room temperature. Tissue sections were incubated in a mixture of 2 primary antibodies in blocking solution (3% BSA, 0.05% Tween20) overnight at 4°C. After washing in PBS, slides were incubated with a mixture of 2 secondary antibodies at a 1:500 dilution in blocking solution (Alexa Fluor 568– conjugated or Alexa Fluor 488–conjugated antibody, Invitrogen). Stained tissue sections were mounted using Prolong Gold Antifade reagent with DAPI (Life Technologies) before being sequentially scanned at a *20x* magnification under a fluorescent microscope (Zeiss). Primary antibodies used in the above experiments are listed in Table 1.

Supplementary Methods References

1. Wershof, E. *et al.* A FIJI macro for quantifying pattern in extracellular matrix. *Life Sci Alliance* **4**, (2021).

2. Birnbaum, D. J., Finetti, P., Birnbaum, D., Mamessier, E. & Bertucci, F. XPO1 Expression Is a Poor-Prognosis Marker in Pancreatic Adenocarcinoma. *J Clin Med* **8**, (2019).

3. Martinez-Useros, J., Martin-Galan, M. & Garcia-Foncillas, J. The match between molecular subtypes, histology and microenvironment of pancreatic cancer and its relevance for chemoresistance. *Cancers (Basel)* **13**, 1–18 (2021).

4. Zhang, S. *et al.* Single cell transcriptomic analyses implicate an immunosuppressive tumor microenvironment in pancreatic cancer liver metastasis. *Nat Commun* **14**, 5123 (2023).

5. Wolf, F. A., Angerer, P. & Theis, F. J. SCANPY: large-scale single-cell gene expression data analysis. *Genome Biol* **19**, 15 (2018).

6. Ge, F. *et al.* Cancer-associated fibroblasts drive early pancreatic cancer cell invasion via the SOX4/MMP11 signalling axis. *Biochim Biophys Acta Mol Basis Dis* **1870**, 166852 (2024).

7. Badia-I-Mompel, P. *et al.* decoupleR: ensemble of computational methods to infer biological activities from omics data. *Bioinformatics advances* **2**, vbac016 (2022).

8. Frankenfield, A. M., Ni, J., Ahmed, M. & Hao, L. Protein Contaminants Matter: Building Universal Protein Contaminant Libraries for DDA and DIA Proteomics. *J Proteome Res* **21**, 2104–2113 (2022).

9. Team, R. C. A Language and Environment for Statistical Computing, R Foundation for Statistical Computing. https://www.r-project.org/ (2024).

10. Blighe, K., Rana, S. & Lewis, M. EnhancedVolcano: Publication-ready volcano plots with enhanced colouring and labeling. (2024).

11. Gu, Z., Eils, R. & Schlesner, M. Complex heatmaps reveal patterns and correlations in multidimensional genomic data. *Bioinformatics* **32**, 2847–2849 (2016).

12. Gu, Z. Complex heatmap visualization. *iMeta* **1**, e43 (2022).

13. Xu, S. *et al.* Using clusterProfiler to characterize multiomics data. *Nat Protoc* **19**, 3292–3320 (2024).

14. Krzywinski, M. *et al.* Circos: an information aesthetic for comparative genomics. *Genome Res* **19**, 1639–1645 (2009).

Figure S1.
Hussain et al.

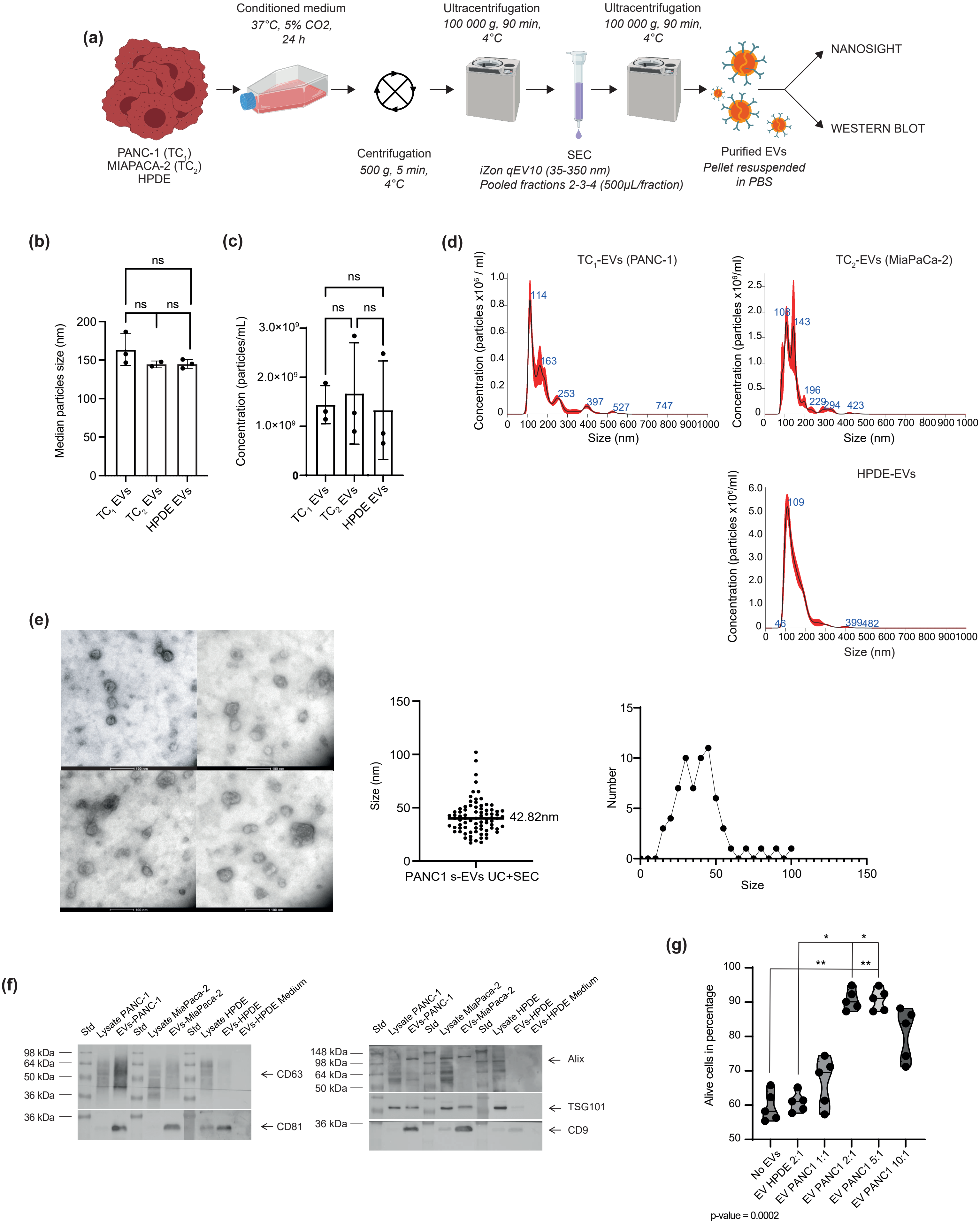

Figure S2.
Hussain et al.

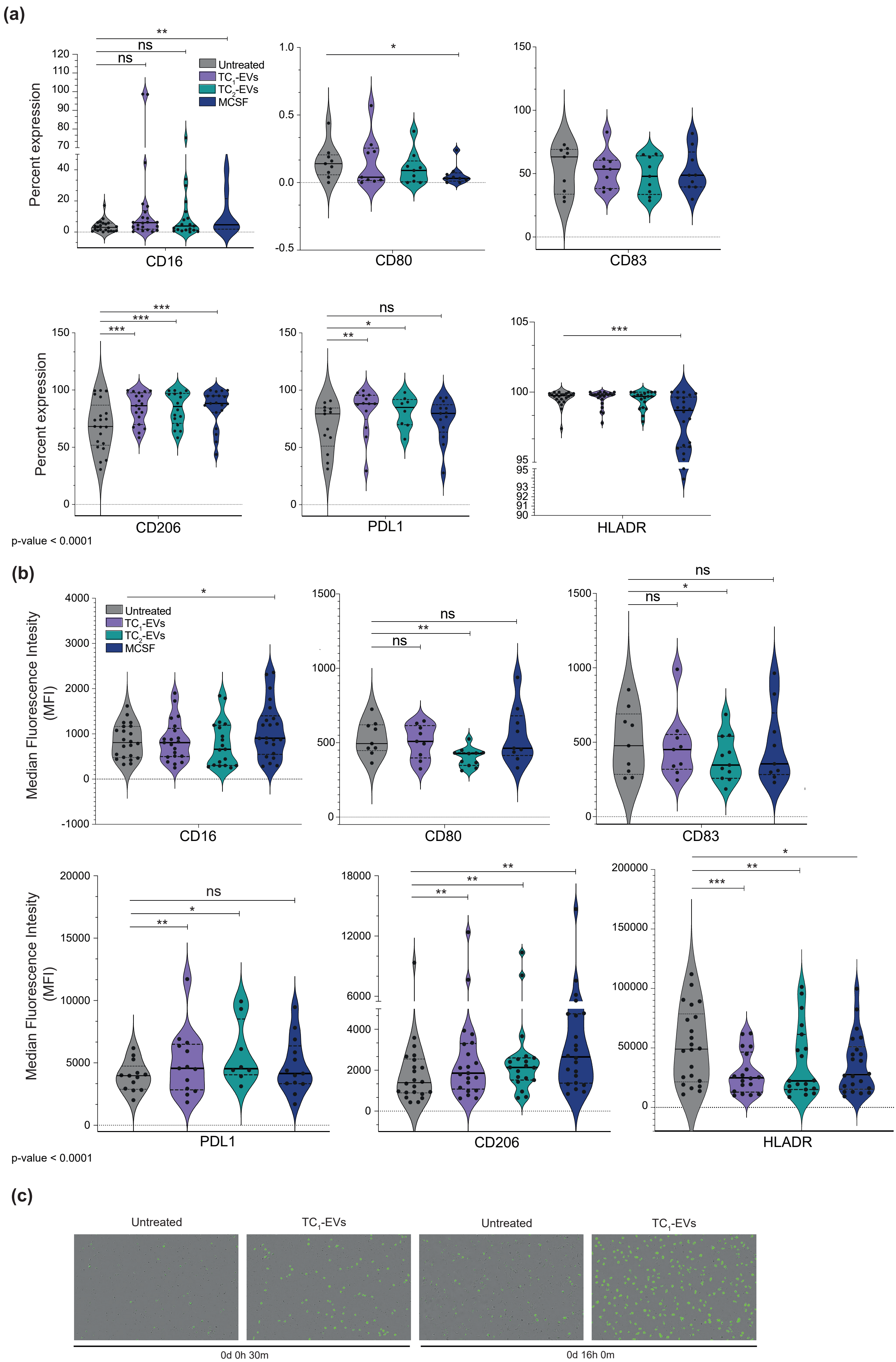

Figure S3.
Hussain et al.

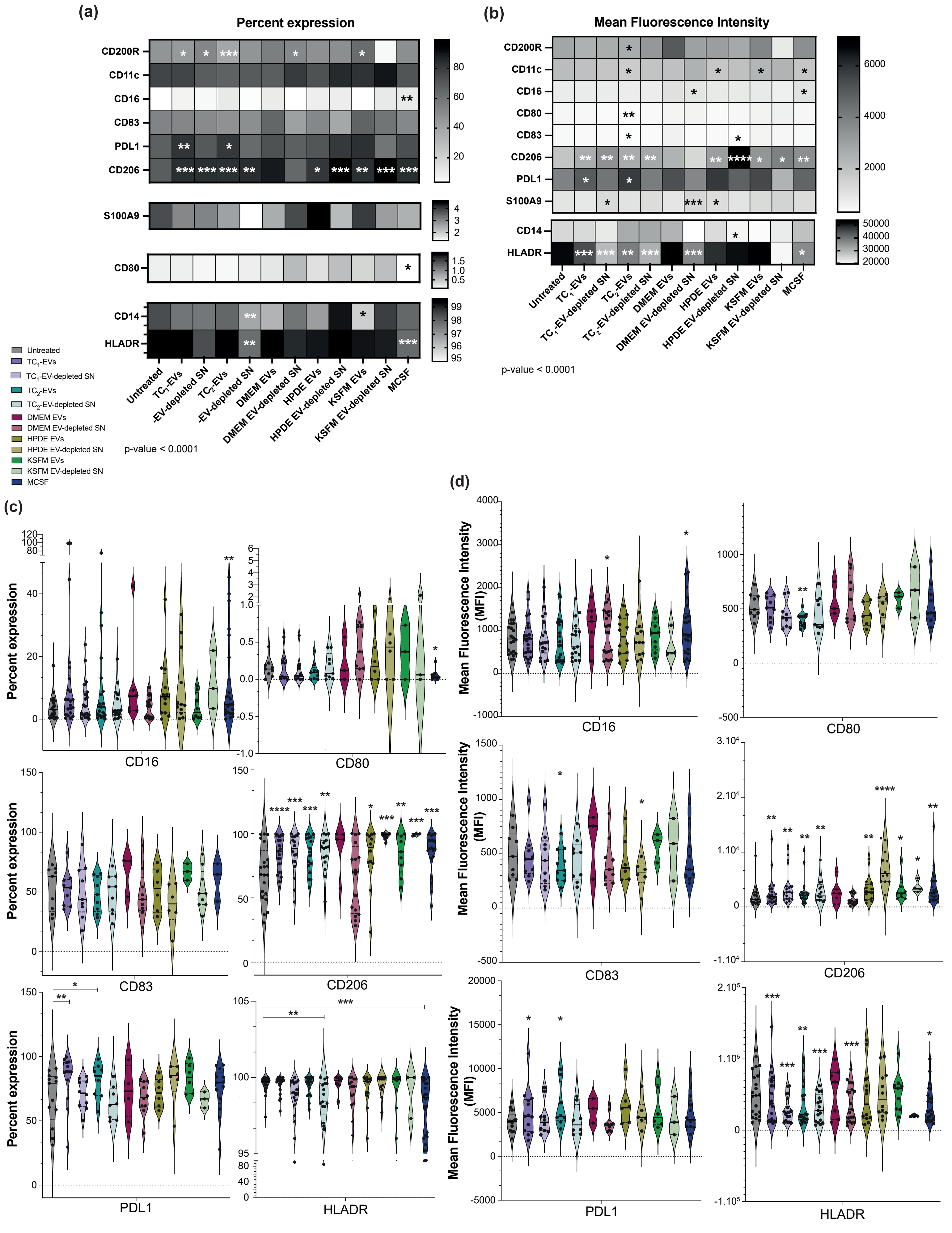

Figure S4.
Hussain et al.

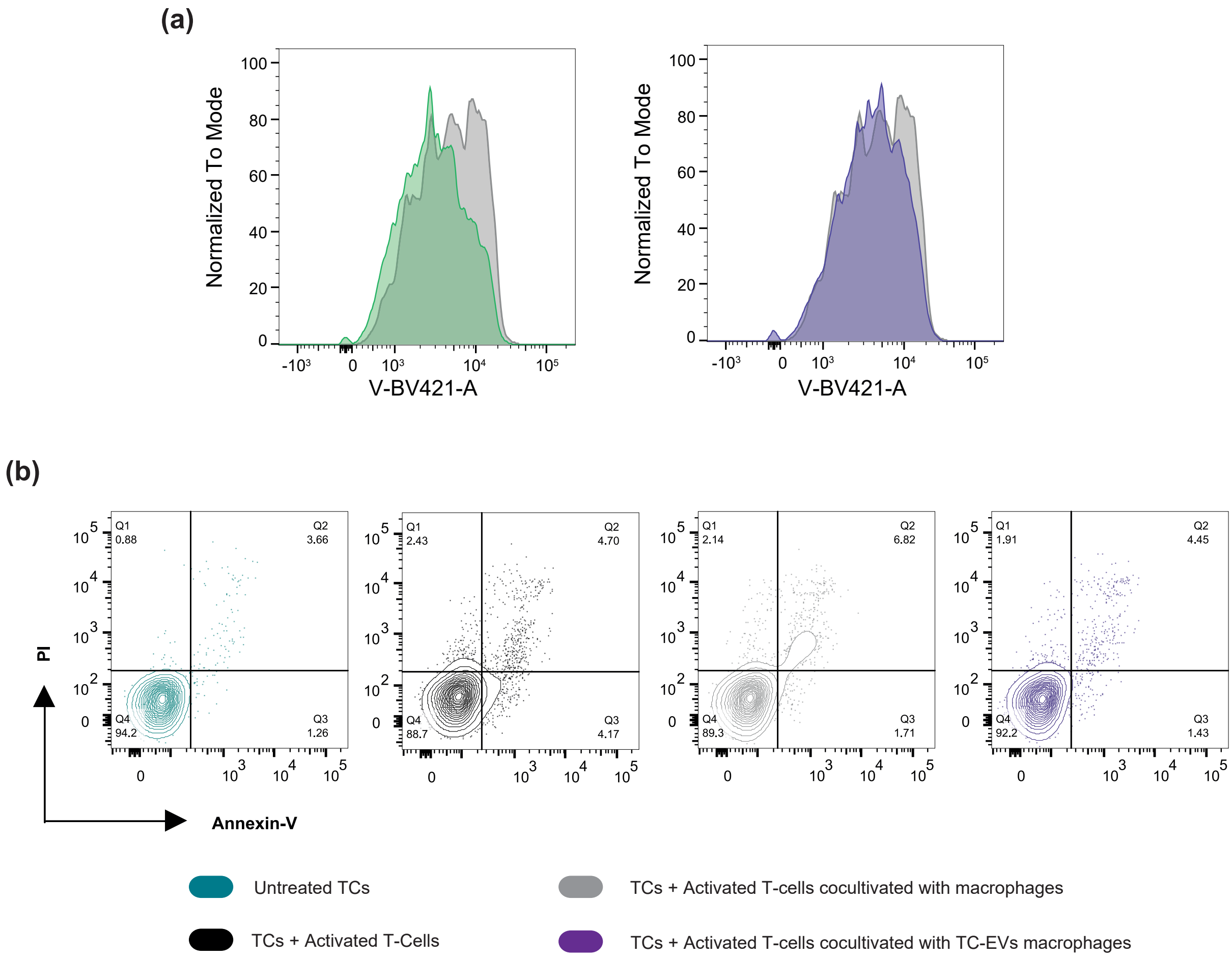

Figure S5.
Hussain et al.

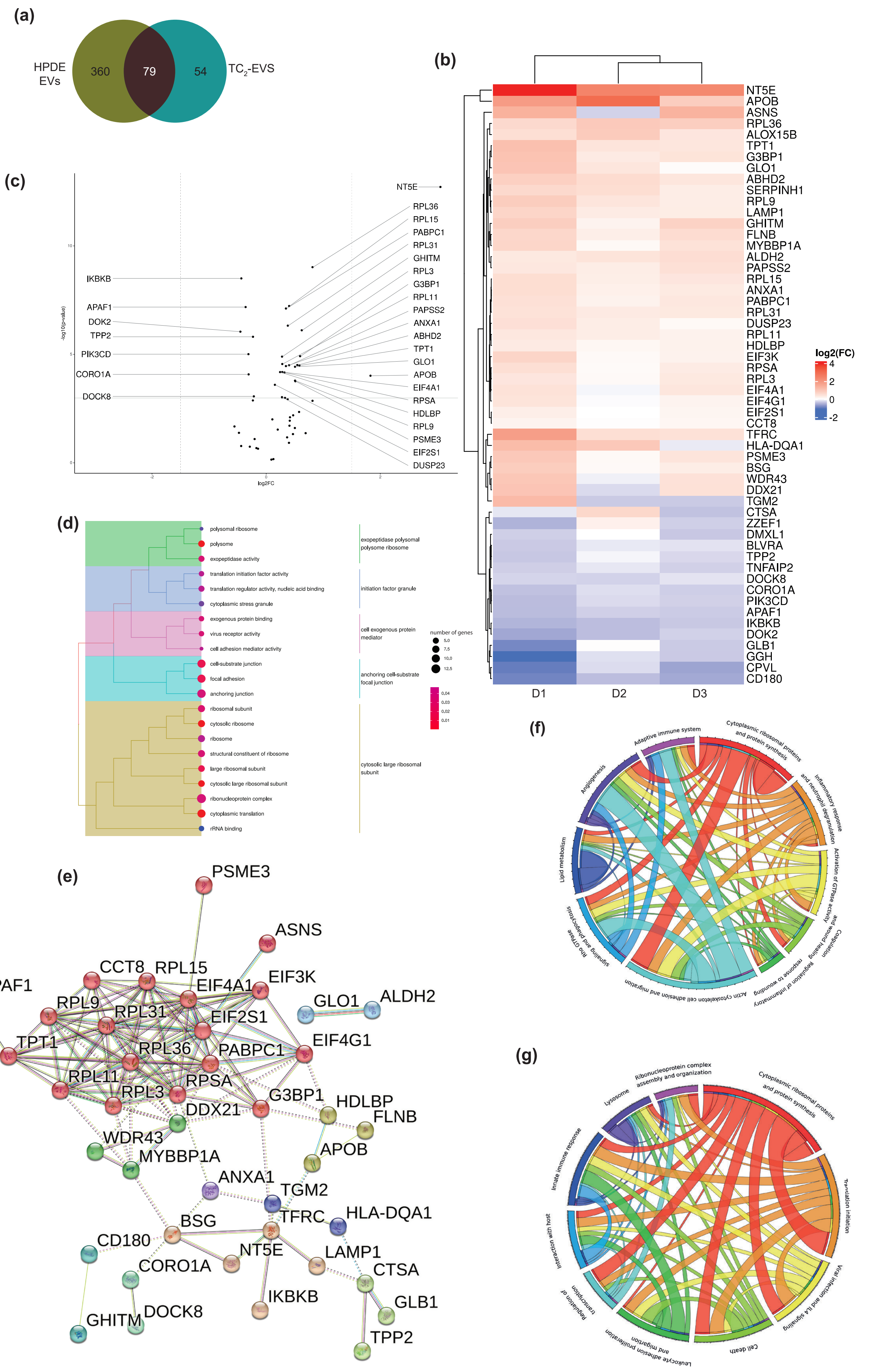

Figure S6.
Hussain et al.

(a)

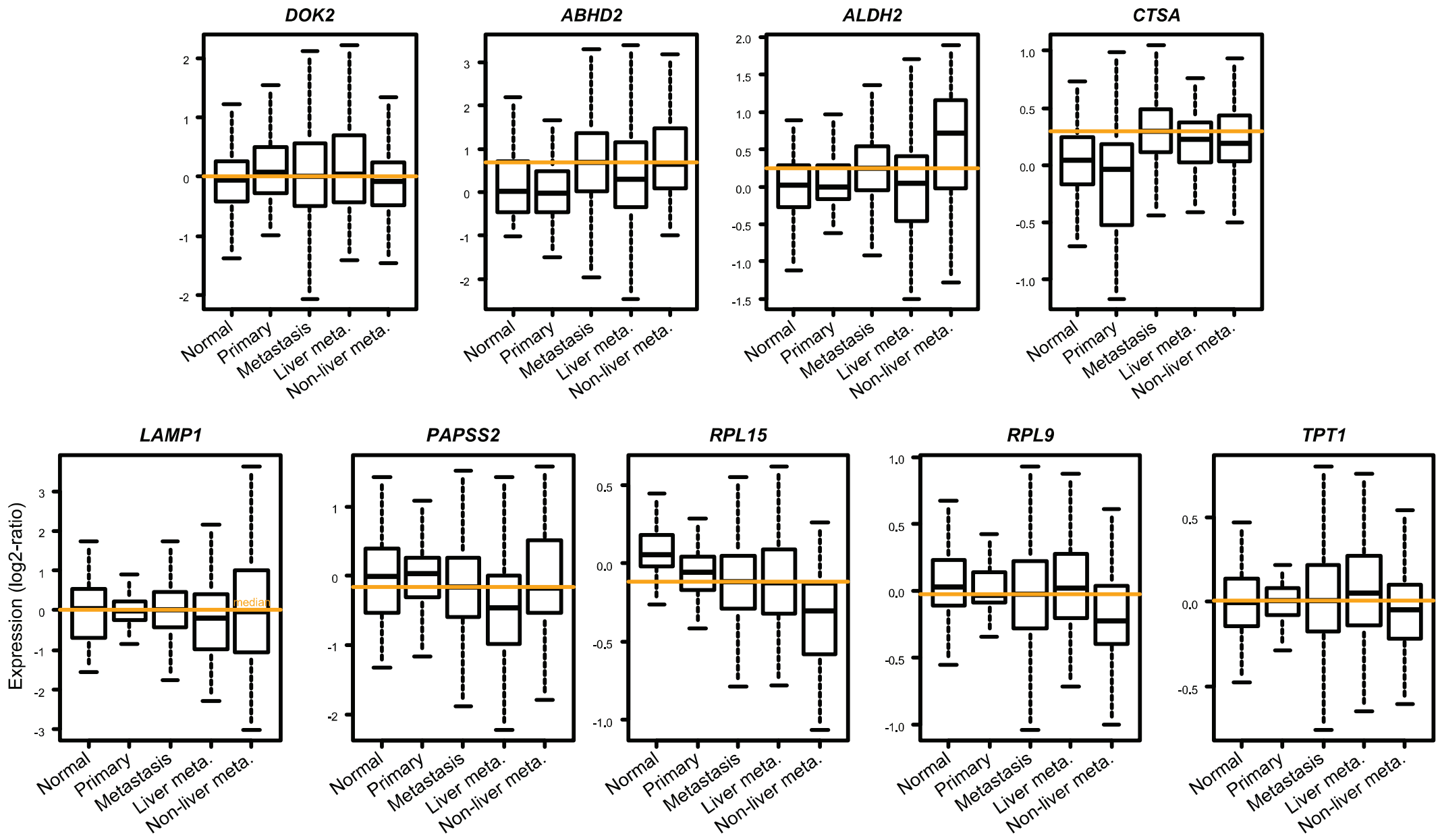

(b)

		N	Primary	Liver meta	Non-liver meta	Primary vs. Metastasis p-value	t	Primary vs. Liver metastasis p-value	t
DOK2	low	560	403 (50%)	31 (56%)	47 (48%)	0.834		0.439	
	high	555	403 (50%)	24 (44%)	50 (52%)				
ABHD2	low	683	469 (50%)	30 (55%)	59 (61%)	0.062		0.605	
	high	565	469 (50%)	25 (45%)	38 (39%)				
ALDH2	low	661	469 (50%)	18 (33%)	62 (64%)	0.607		**1.87E-02**	
	high	587	469 (50%)	37 (67%)	35 (36%)				
CTSA	low	703	469 (50%)	32 (58%)	65 (67%)	**2.10E-03**		0.298	
	high	545	469 (50%)	23 (42%)	32 (33%)				
LAMP1	low	638	469 (50%)	28 (51%)	59 (61%)	0.117		1.000	
	high	610	469 (50%)	27 (49%)	38 (39%)				
PAPSS2	low	624	469 (50%)	28 (51%)	67 (69%)	**5.55E-03**		1.000	
	high	624	469 (50%)	27 (49%)	30 (31%)				
RPL15	low	604	469 (50%)	41 (75%)	51 (53%)	**2.03E-02**		**6.72E-04**	
	high	644	469 (50%)	14 (25%)	46 (47%)				
RPL9	low	620	469 (50%)	37 (67%)	42 (43%)	0.716		**1.87E-02**	
	high	628	469 (50%)	18 (33%)	55 (57%)				
TPT1	low	626	469 (50%)	32 (58%)	43 (44%)	0.950		0.298	
	high	622	469 (50%)	23 (42%)	54 (56%)				

(c)

			Primary	Liver meta	Non-liver meta	Primary vs. Metastasis p-value	t	Primary vs. Liver metastasis p-value	t
ALOX15B	low	590	469 (50%)	15 (27%)	28 (29%)	**1.02E-06**		**1.70E-03**	
	high	658	469 (50%)	40 (73%)	69 (71%)				
DOCK8	low	649	457 (50%)	42 (76%)	51 (53%)	**1.36E-02**		**2.52E-04**	
	high	575	457 (50%)	13 (24%)	46 (47%)				
RPL11	low	632	469 (50%)	46 (84%)	60 (62%)	**9.26E-06**		**2.44E-06**	
	high	616	469 (50%)	9 (16%)	37 (38%)				

(d)

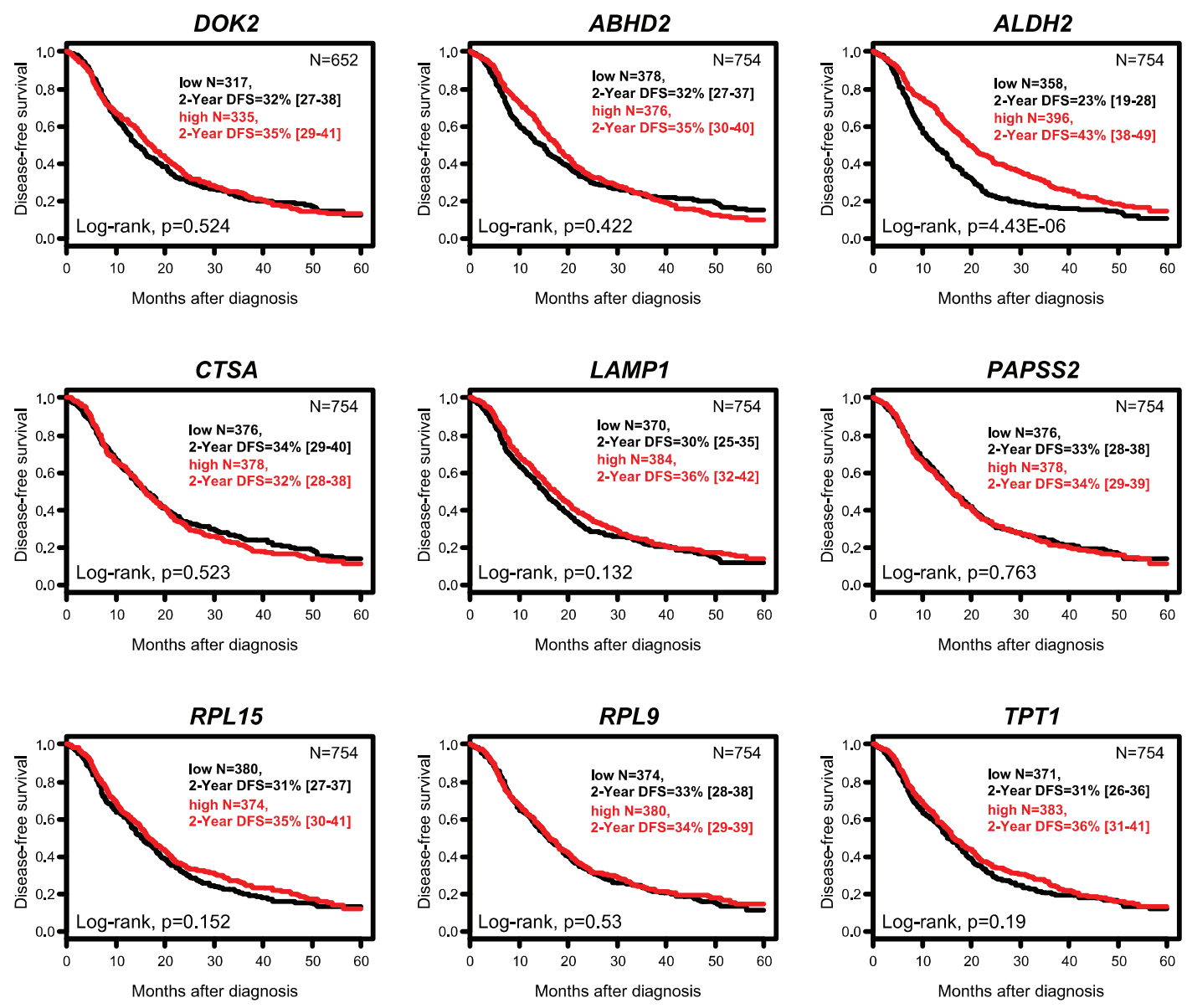

(e)

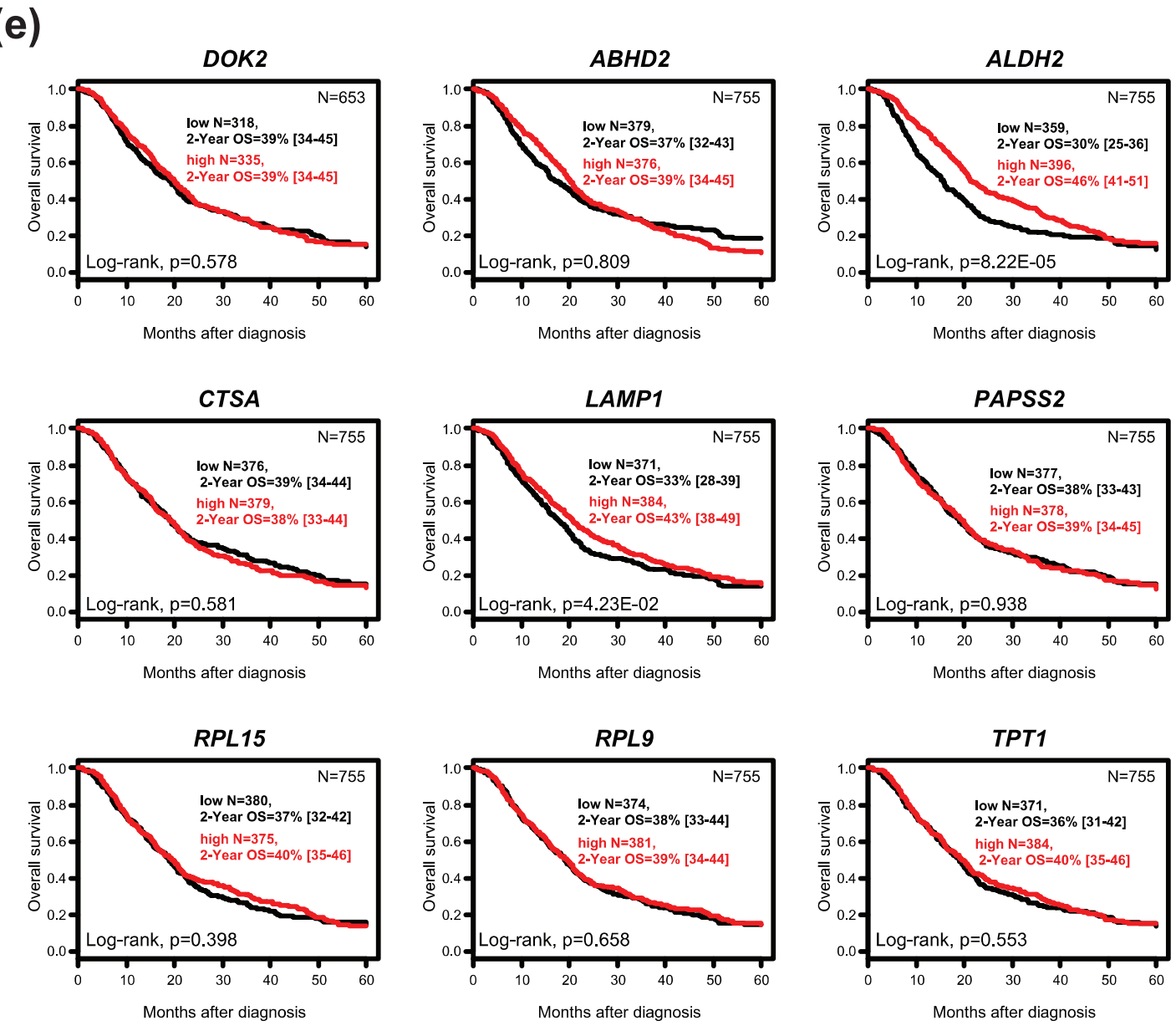

(f)

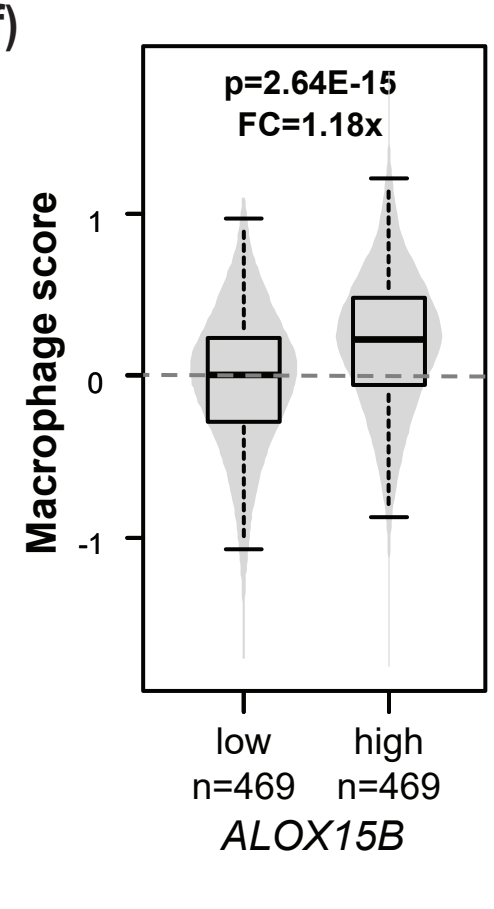

(g)

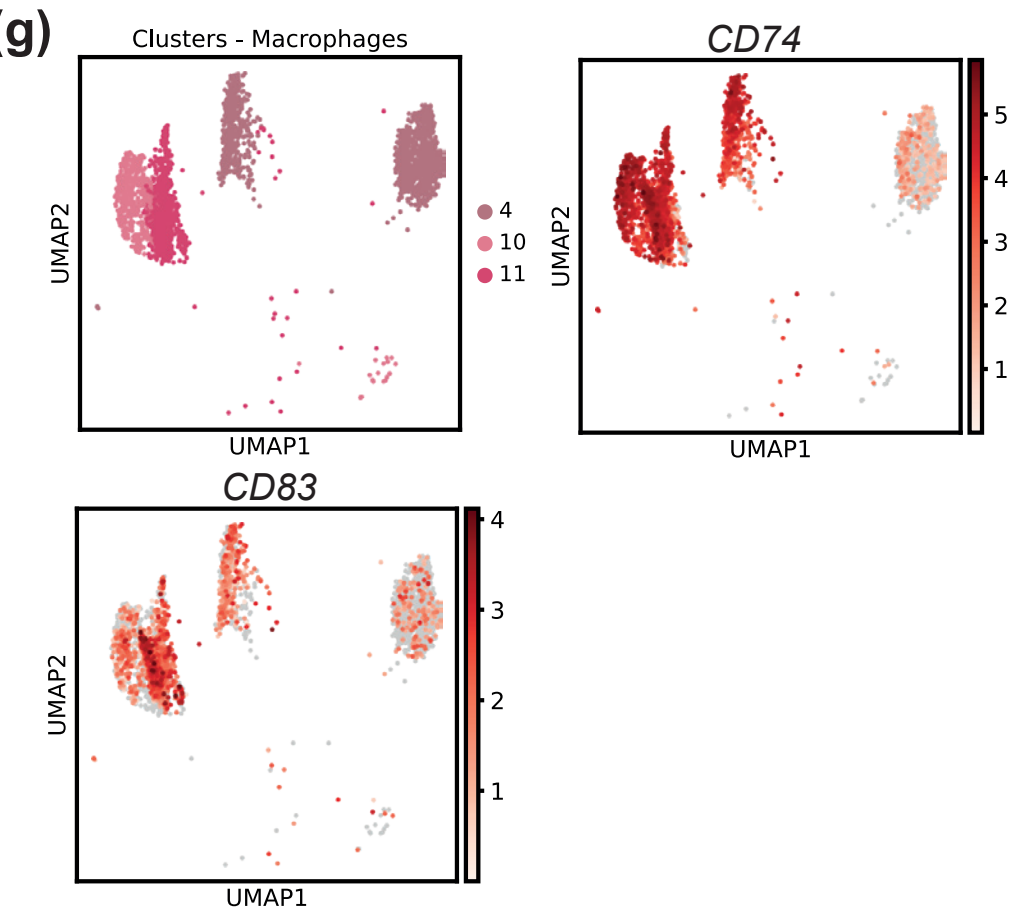

(h)

Cox, models comparison (OS) Likelihood (X² Chi-squared)		
ALOX15B	LRX²	14.36
	p-value	1.51E-04
Macrophage score (Bindea)	LRX²	10.61
	p-value	1.12E-03
ALOX15B+macrophage	LRX²	19.96
	p-value	4.63E-05
ALOX15B	**ΔLRX²**	**5.60**
vs. ALOX15B+macrophage	**p-value**	**1.80E-02**
macrophage	**ΔLRX²**	**9.35**
vs. ALOX15B+macrophage	**p-value**	**2.23E-03**

Figure S7.
Hussain et al.

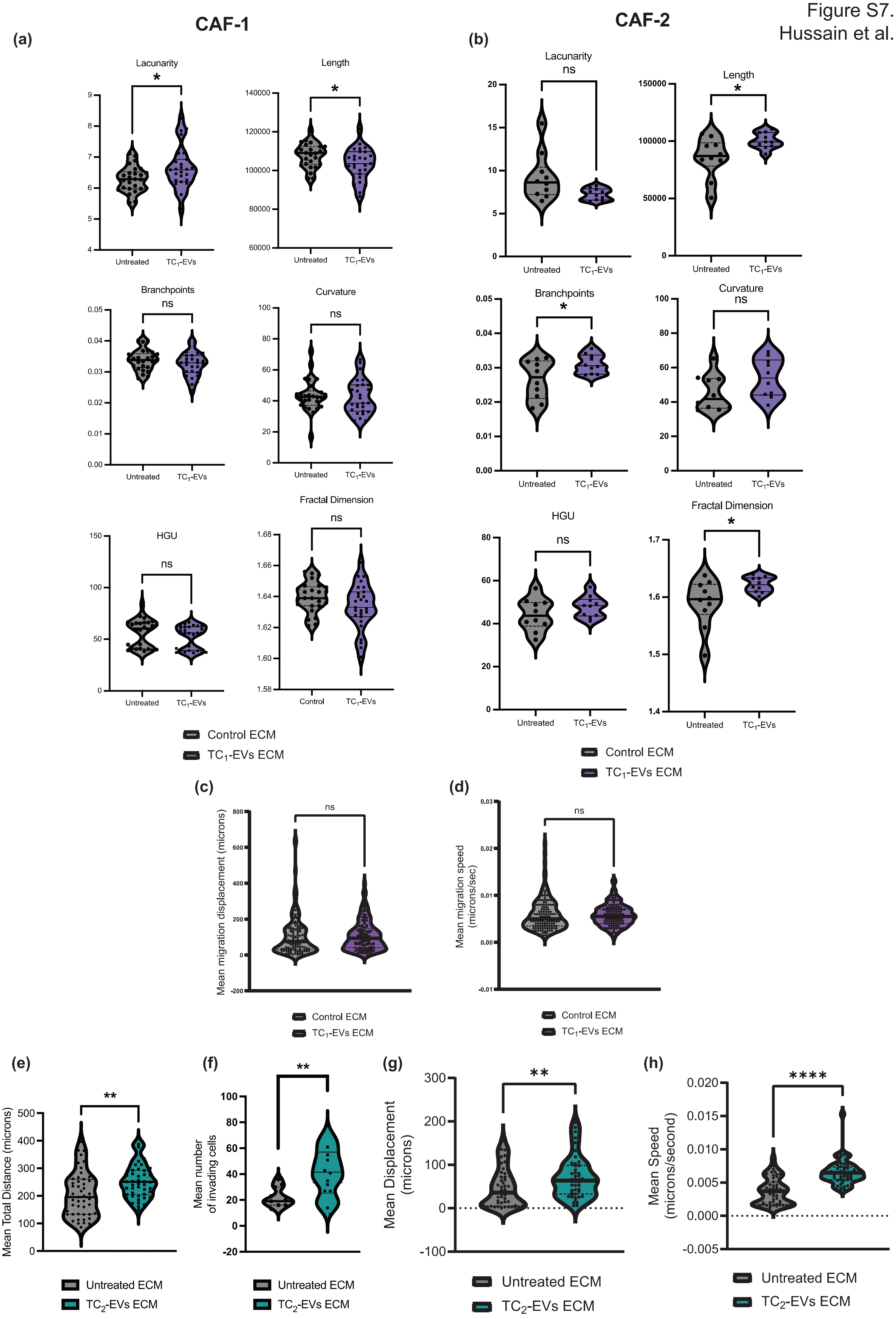

Figure S8.
Hussain et al.

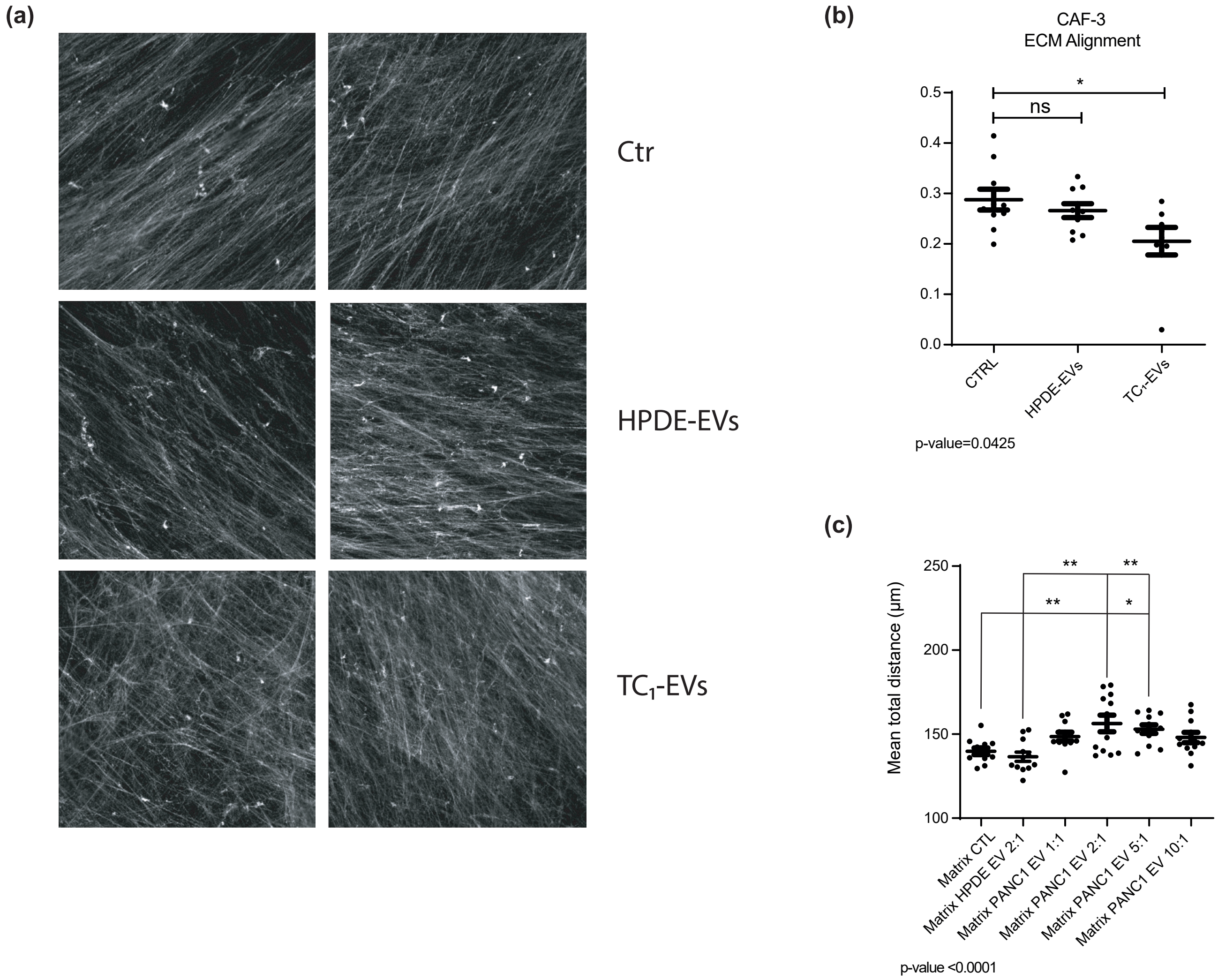

Figure S9.
Hussain et al.

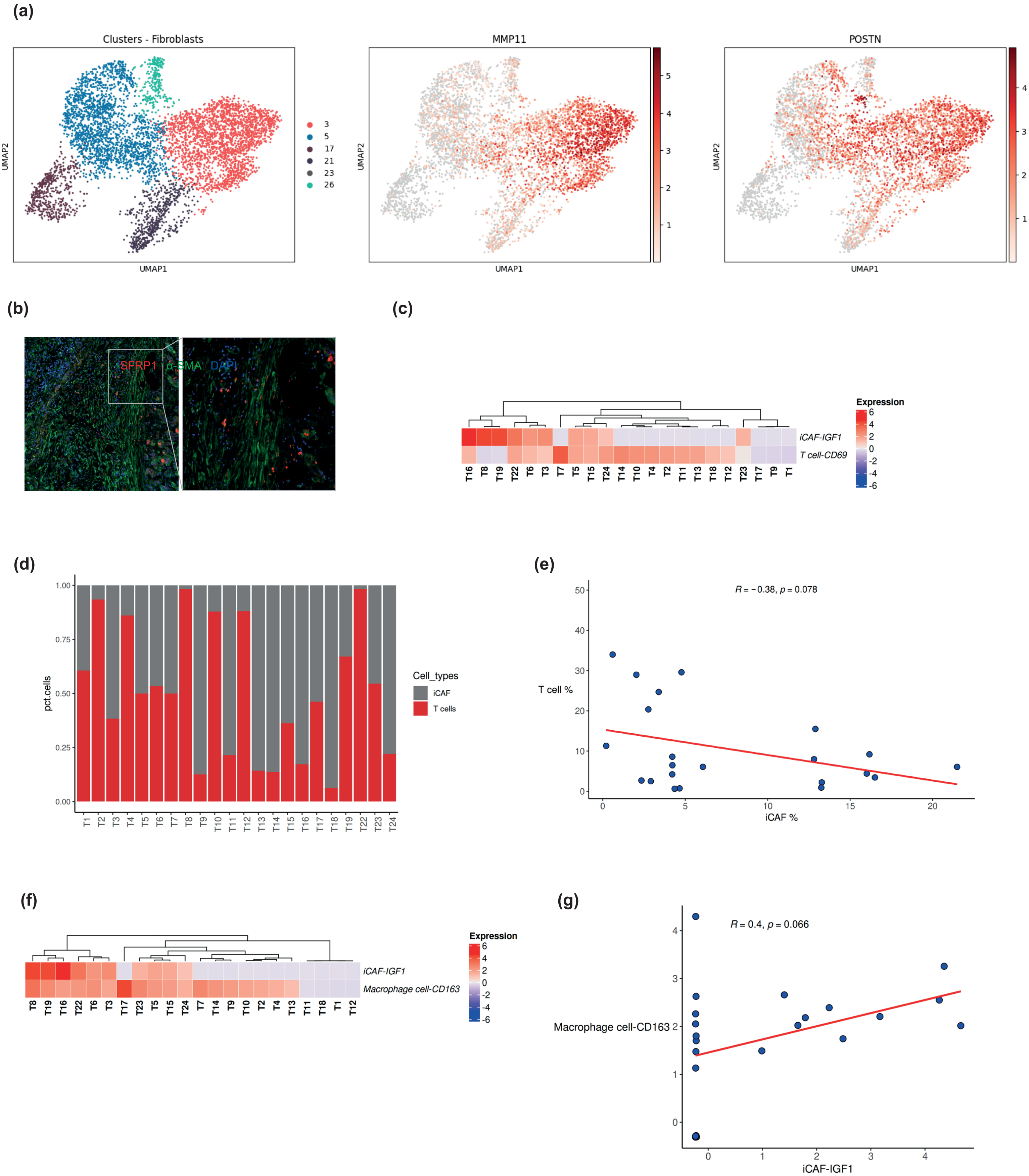

Supplement: Supplementary file 1 [file jci-136-197733-s172.pdf]
